# A nano phototheranostic approach of toluidine blue conjugated gold silver core shells mediated photodynamic therapy to treat diabetic foot ulcer

**DOI:** 10.1038/s41598-021-04008-x

**Published:** 2021-12-27

**Authors:** Farheen Akhtar, Asad U. Khan, Bushra Qazi, Senthilguru Kulanthaivel, Prashant Mishra, Kafil Akhtar, Asif Ali

**Affiliations:** 1 Medical Microbiology and Molecular Biology Lab., Interdisciplinary Biotechnology Unit, Aligarh Muslim University, Aligarh, 202002 UP India; 2 Department of Biochemical Engineering and Biotechnology, Indian Institute of Technology , Delhi, India; 3 Department of Pathology, JNMC, A.M.U., Aligarh, India; 4 Department of Biochemistry, F/O Medicine, JNMC, A.M.U., Aligarh, India

**Keywords:** Biochemistry, Immunology, Microbiology, Health care

## Abstract

Diabetic foot infection caused by multidrug-resistant bacteria, is becoming serious problem. Moreover, polymicrobial biofilms contribute significantly to the persistent infections. In the present study, we investigated the effectiveness of novel toluidine blue conjugated chitosan coated gold–silver core–shell nanoparticles (TBO–chit–Au–AgNPs) mediated photodynamic therapy and demonstrate their use as a nontoxic antibacterial therapy to combat diabetic foot ulcer (DFU) caused by multi-drug resistant strains both in monomicrobial and polymicrobial state of infection. In vitro efficacy of TBO–chit–Au–AgNPs mediated photodynamic therapy (PDT) against polymicrobial biofilms was determined using standard plate count method and compared with that of monomicrobial biofilms of each species. Different anti-biofilm assays and microscopic studies were performed to check the efficacy of TBO–chit–Au–AgNPs mediated PDT, displayed significant decrease in the formation of biofilm. Finally, its therapeutic potential was validated in vivo type-2DFU. Cytokines level was found reduced, using nano-phototheranostic approach, indicating infection control. Expression profile of growth factors confirmed both the pathogenesis and healing of DFU. Hence, we conclude that TBO–chit–Au–AgNPs mediated PDT is a promising anti-bacterial therapeutic approach which leads to a synergistic healing of DFU caused by MDR bacterial strains.

## Introduction

Type 2 diabetes mellitus (DM) has become an utmost health concern which comprised of 90–95% diabetes among worldwide population^1^. Nevertheless, immunocompromised patients identified with diabetes mellitus are more prone to be suffered from non-healing wounds^2^. It has also been reported that up to one-third of people with diabetes may develop foot ulcer during their lifetime and over 50% of these ulcers become infected. This challenge is further aggravated by the emergence of multidrug-resistance (MDR) strains induced diabetic foot infections (DFIs) which leads to increasing morbidity and mortality, and risk of lower extremity amputation (LEA) which causes low quality of life^3–6^. Furthermore, biofilm-forming MDR strains are far more impervious to antimicrobials than organisms in suspension^7–9^. It has been stated earlier that biofilms intricate up to 65% of infections, leading to severe illness with a prolonged stay in hospital settings which may increase cost of treatment as well as mortality rate^10^.

The most commonly found microorganisms which are isolated from patients with diabetic foot ulcer (DFU) were reported as *Pseudomonas aeruginosa*, *Staphylococcus aureus*, *Escherichia coli*,* Enterococcus* spp., *Streptococcus* spp., *Proteus mirabilis* and anaerobes^11^. Among them, *S. aureus* and *P. aeruginosa* are the major cause of DFIs. For instance, polymicrobial (mixed microbial culture) *S. aureus* and *P. aeruginosa* infections appear frequently in deep or chronic wounds^12,13^. The mutualistic and parasitic interactions cause synergistic association among the two species which led to the development of infections^14,15^. Inclusion of mixed microbial species in a single community of biofilms causes several benefits, such as an enlarged gene pool with more effectual DNA sharing, quorum sensing systems, metabolic cooperation, etc^16^. Since no antibiotic is left to treat such infections therefore, the need of an hour is to search for alternative therapy.

**Figure 1:**
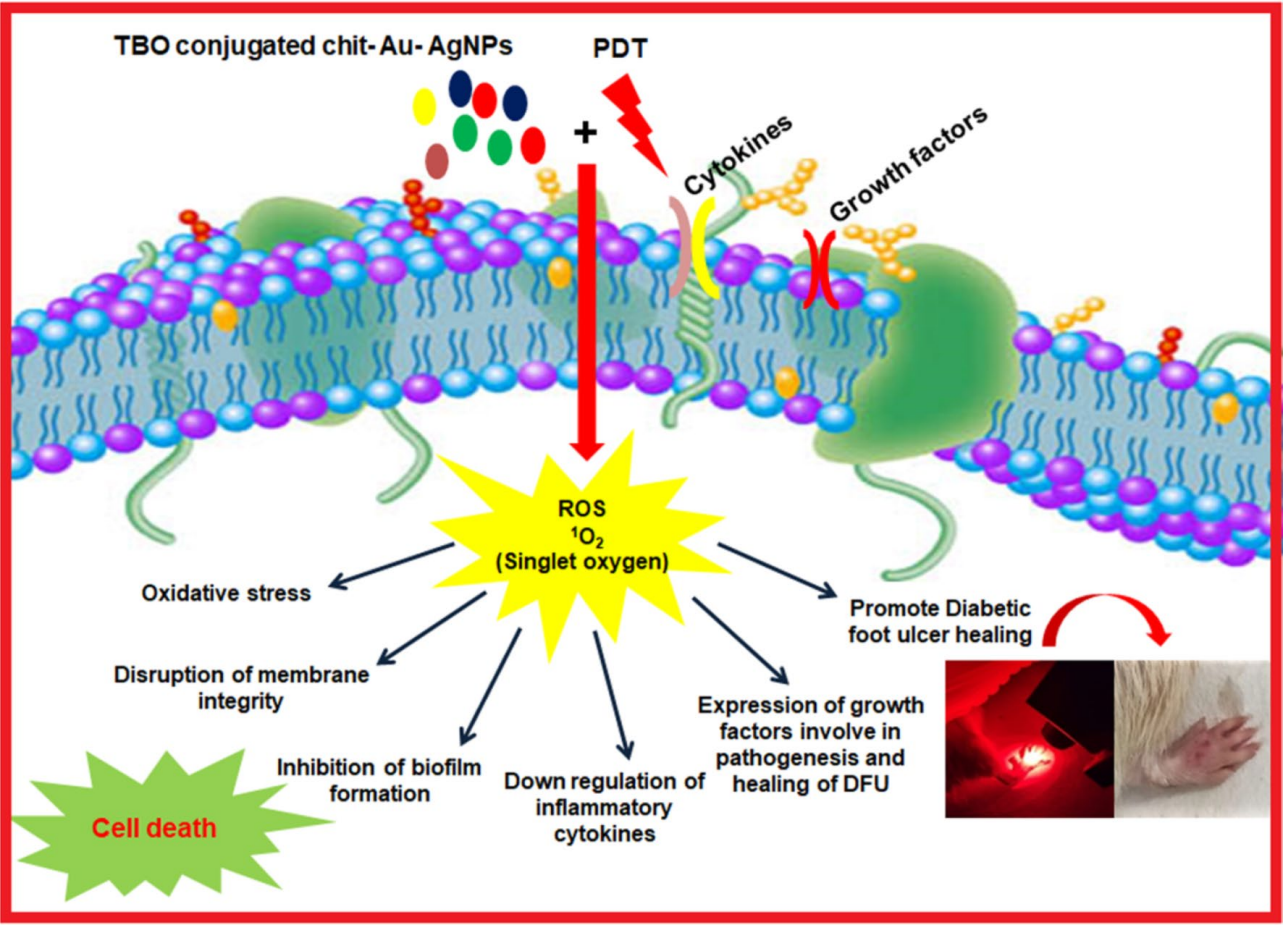
Schematic representation of nanoparticle mediated antimicrobial photodynamic therapy against diabetic foot ulcer.

Metallic nanomaterials have attracted considerable attention to control the spread of infections over the last decade. These nanomaterials in different forms may bind to the cell surface of bacteria, causing membrane damage which in turn leads to an alteration in membrane potential as well as permeability followed by cytoplasmic leakage and cellular damage. In addition, metal nanoparticles produce different kinds of intracellular reactive oxygen species (ROS) which damages microbial membrane and other cellular components, thereby causing cell death^17–19^. The antimicrobial actions of metal nanoparticles including gold and silver have already been established. Gold nanoparticles (AuNPs) have been reported to be used extensively in therapeutics and diagnosis because of their small size and large surface-area-to-volume ratio^20–22^. Here, we initiated to fabricate a chitosan-coated gold nanoparticle (chit-AuNPs) as a core material^23^, followed by the deposition of the silver shell. It is worth to mention that chitosan biopolymer act as an effective reducing and stabilizing agent as well as an external layer to provide the nanocomposites with several advantages^24^. Whereas, silver ions act upon various sites in microorganisms to cease their growth. Previously, it was shown that silver nanoparticles are generally liable for the contact killing of microorganisms^25–30^. In this regard, chitosan coated gold–silver core–shell nanoparticles (chit–Au–AgNPs) would be an efficient antimicrobial agent. Moreover, we intend to conjugate chit-Au–AgNPs metal composite with a photosensitizer (TBO), which will further enhance the antibacterial efficacy of this nanocomposite and possibly eradicates infection caused by resistant bacteria. Toluidine blue O (TBO) is a cationic phenothiazine dye that has been well studied as an antibacterial photosensitizer. It departs a high quantum of cytotoxic singlet oxygen during photosensitization with 630 nm wavelength of light^31–36^. Recently, photodynamic therapy (PDT) has proven as multipronged strategy, which is far most effective to inactivate microorganisms such as bacteria, fungi, viruses and yeast^37–48^, as compared to antibiotics. PDT combines the action of light on a non-toxic photosensitizer (PS) which causes production of reactive oxygen species being toxic to cellular components. This approach is highly effective in killing MDR strains since microorganisms do not acquire resistance toward PDT^49^.The use of TBO–chit–Au–AgNPs mediated PDT has not been reported yet. Therefore, we initiated this study to provide a novel approach toward potential applications of nano-phototheranostic complex, as a nontoxic antibacterial agent to combat DFU caused by multi-drug resistant bacterial strains (Fig. 1).

## Results

### Synthesis and characterization of TBO–chit–Au–AgNPs

**Figure 2:**
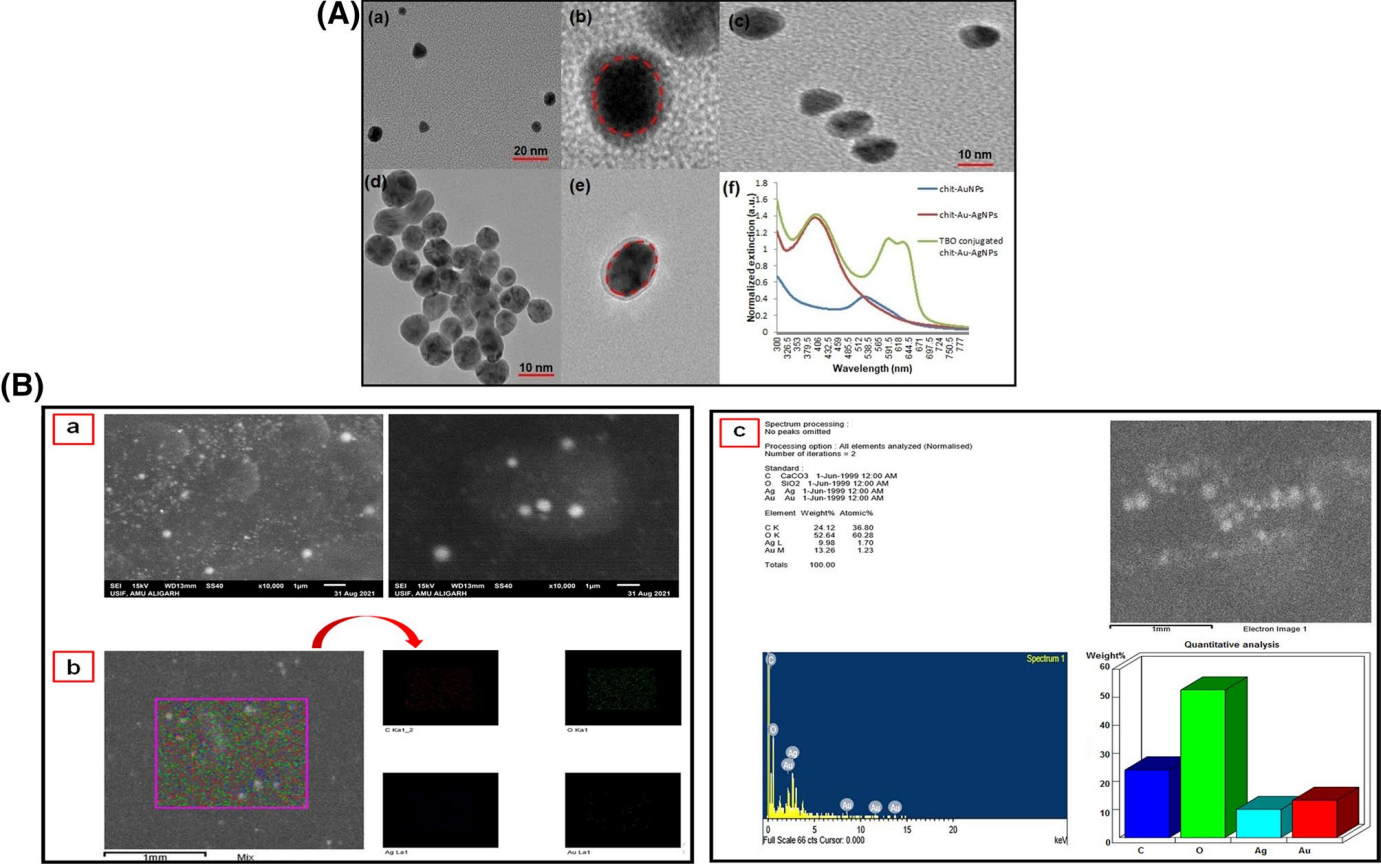
Characterization of nanoparticles: (**A**) (a) TEM image of spherical chit–AuNPs. (b)–(d) TEM images of chit–Au–AgNPs after additions of AgNO_3_ aliquots. The interface between the Au–core and the Ag–shell was marked by a red dashed line in (**A**(b)). (e) A closer view of an Au–AgNP coated by a chitosan layer. (f) UV–Vis absorption spectra of chit–AuNPs, chit–Au–AgNPs and TBO–chit–Au–AgNPs. (**B**) (a) Scanning electron microscopy (SEM) images of chit–Au–AgNPs, (b) Elemental distribution of Au, Ag, C and O in the sample (chit–Au–AgNPs) from Energy dispersive X-ray spectrometry (EDS) analysis, (c) EDS profile of chit–Au–AgNPs and quantitative analysis of Au, Ag, C and O in the sample.

Chitosan–coated gold nanoparticles (chit–AuNPs, Fig. 2A(a)) were synthesized as core materials instead of citrate or dextran capped gold nanoparticles to produce biocompatible gold–silver core–shell nanoparticles (chit–Au–AgNPs). The synthesized TBO–chit–Au–AgNPs were characterized by UV-spectroscopy, TEM, DLS and Zeta potential. Furthermore, we have performed elemental mapping of chit–Au–AgNPs using scanning electron microscopy (SEM) with energy dispersive X-ray spectrometry (EDS). The addition of TBO with chit–Au–AgNPs under dark conditions has led to the formation of TBO–chit–Au–AgNPs (Fig. 2A(f)-green spectrum). The UV-visible spectrum of the synthesized chit–AuNPs (Fig. 2A(f)-blue spectrum) exhibits a well-defined SPR (surface plasmon resonance) band centered at 530 nm. Thereafter, AgNO_3_ aliquots were added into a solution containing colloidal chit-AuNPs and ascorbic acid, changes the SPR extinction spectrum. For instance, after 60 min, occurrence of a new band takes place at 405 nm (Fig. 2A(f)-red spectrum) upon first addition of AgNO_3_ aliquots. The morphological progression of chit–Au–AgNPs was further confirmed by TEM. The dark gold core and the brighter silver shell as observed in Fig. 2A(b–d), was clearly distinguishable because of higher electronic density of gold than silver. Absence of free AgNPs in TEM images illustrated the stepwise addition of AgNO_3_, leading to the formation of bimetallic nanoparticles than monometallic. Furthermore, Fig. 2A(e) showed the presence of chitosan layer as a faint shadow which encapsulates the Au–AgNPs after their formation. Moreover, the result of scanning electron microscopy (SEM) with energy dispersive X-ray spectrometry (EDS) analysis (EDX) clearly showed the presence of Au, Ag and chitosan in pure form with weights of 13.26% Au, 9.98% Ag, 24.12% C and 52.64% O in the sample (Fig. 2B-a, b and c).The average size of chit–AuNPs, chit–Au–AgNPs and TBO–chit–Au–AgNPs were found to be 129.4 nm, 131 nm, and 134 nm, respectively, as analyzed by dynamic light scattering (DLS) (Table 1). Changes in the polydispersity index (PDI) were also measured over this time. The PDI of chit-AuNPs, chit–Au–AgNPs and TBO–chit–Au–AgNPs remained low, indicating that there is least or no aggregation of particles. In addition, the result of zeta potential clearly illustrates chit–Au–AgNPs synthesized through stepwise addition of AgNO_3_ retains their positive zeta potential of +37.6 mV, which shows that the polymeric coating is well-preserved all through the formation of core–shell nanoparticles (chit–Au–AgNPs). Even after the addition of TBO, chit–Au–AgNPs exhibited strong positive zeta potential of +41.2 mV, suggesting the stability and biocompatibility of synthesized TBO–chit–Au–AgNPs (Table 1).

**Table 1:** Hydrodynamic diameter (size by number) obtained from DLS, Polydispersity index (PDI) and zeta potential values of chit–AuNPs, chit–Au–AgNPs and TBO–chit–Au–AgNPs.

Sample	Size (d, nm)	Polydispersity index (PDI)	Zeta potential (mV)
Chit–AuNPs	129.4	0.107	49
Chit–Au–AgNPs	131	0.067	37.6
TBO–chit–Au–AgNPs	134	0.085	41.2

### Effect of TBO–chit–Au–AgNPs mediated photodynamic therapy on cellular toxicity

**Figure 3:**
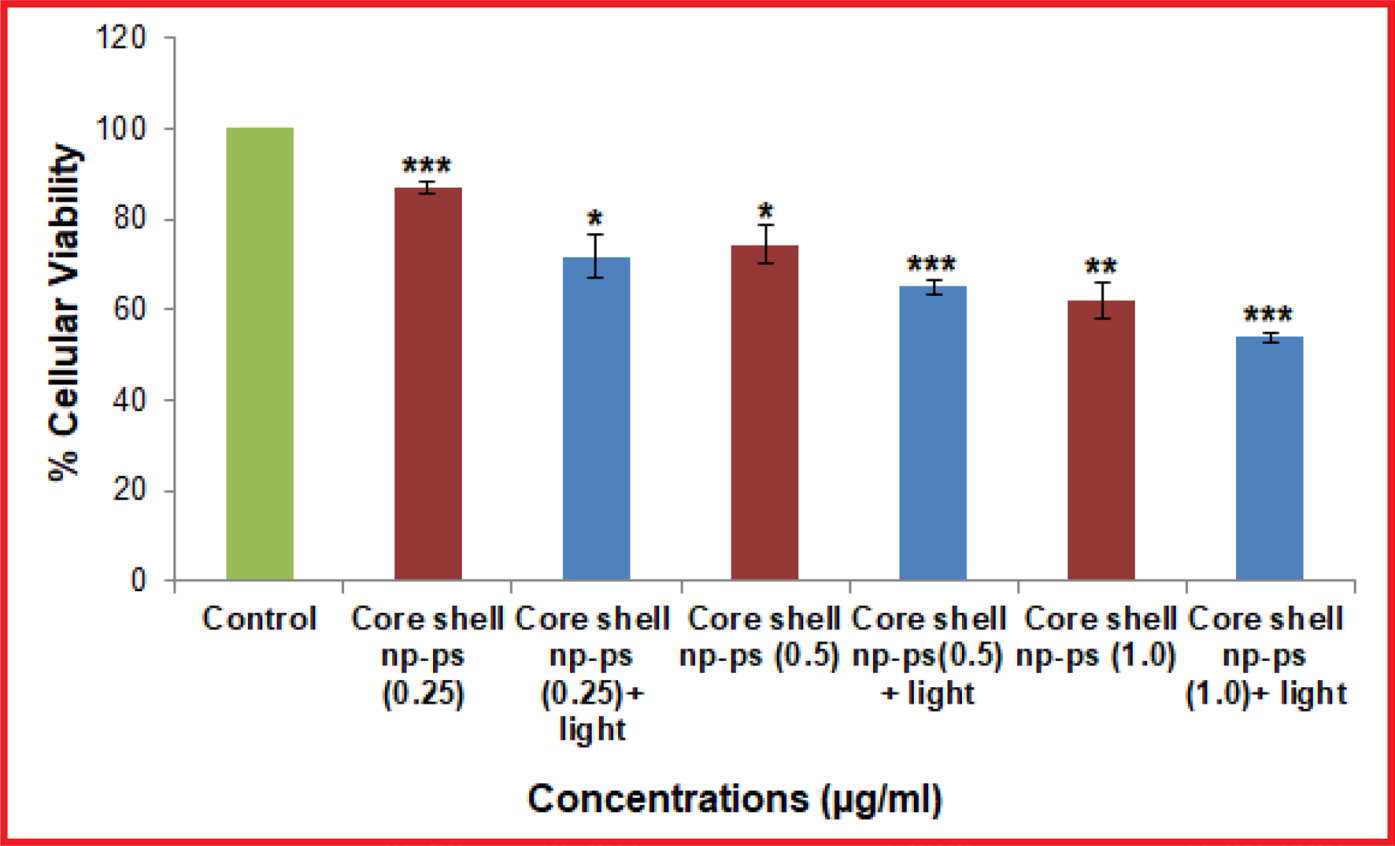
Cytotoxicity assay: Percentage viable cells as measured after 24 h, after treatment with TBO–chit–Au–AgNPs in absence and presence of laser irradiation. Data expressed as mean ± SD, n = 3 (*p*-value *p < 0.01; **p < 0.001, ***p < 0.0001, ns = not significant).

The relative cellular viability of L929 fibroblast cells in the presence of various concentrations (0.25 mM, 0.5 mM and 1 mM) of TBO–chit–Au–AgNPs with and without laser irradiation is shown in Fig. 3. Almost 65.12% and 74.32% viability were observed at our tested concentration (0.5 mM) with and without laser irradiation respectively. Thus, the concentration of the TBO–chit–Au–AgNPs and exposure time (100 J/cm^2^) of red laser light used in this study was found to be non-toxic.

### In vitro anti-bacterial activity of TBO–chit–Au–AgNPs mediated photodynamic therapy on monomicrobial and polymicrobial biofilms of *S. aureus* and *P. aeruginosa*

**Figure 4:**
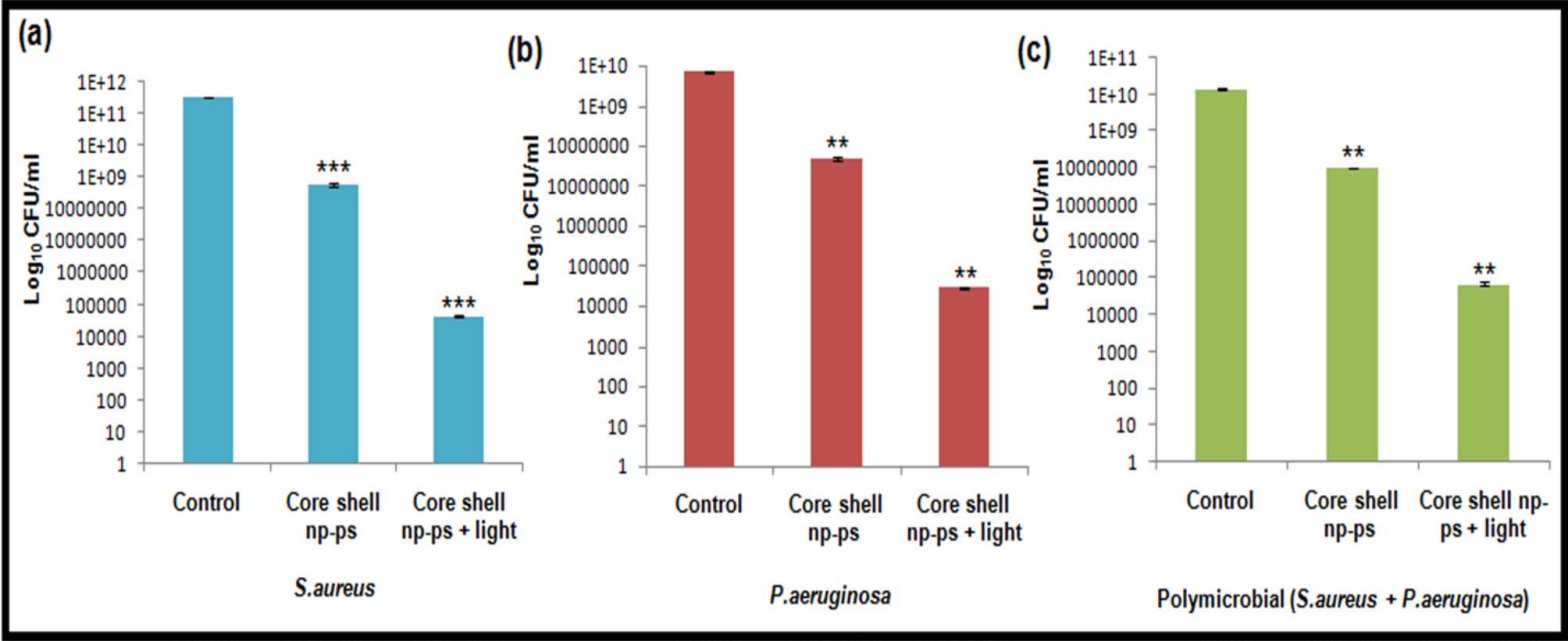
In vitro colony formation: (**a**) Monomicrobial *S. aureus*, (**b**) Monomicrobial *P. aeruginosa *and, (**c**) Polymicrobial *S. aureus* + *P. aeruginosa* colonies after incubation with TBO–chit–Au–AgNPs, TBO–chit–Au–AgNPs followed by irradiation with 630 nm laser for 12 min and 50 s which corresponds to 100 J/cm^2^ at 0.1300 W/cm^2^. Data are presented as mean ± SD (n = 3) and normalized to that of untreated control. Three replicates were performed for each experiment. Statistical significance was determined using one-way analysis of variance (*p*-value *p < 0.01, **p < 0.001, ***p < 0.0001, ns = not significant).

The antibacterial activity of TBO–chit–Au–AgNPs in the presence as well as in the absence of laser irradiation was assessed using colony formation assay against polymicrobial biofilms and compared with that of monomicrobial biofilms. After incubation for 24 h, the resulting monomicrobial biofilms showed 2.76 log_10_ CFU/mL reduction of *S.aureus* and 2.17 log_10_ CFU/mL reduction of *P. aeruginosa* in the group treated with TBO–chit–Au–AgNPs only. While, 2.15 log_10_ CFU/mL reduction was seen in polymicrobial biofilms (Fig. 4). A substantial reduction in bacterial load was achieved when monomicrobial and polymicrobial biofilms of *S. aureus* and *P. aeruginosa* treated with TBO–chit–Au–AgNPs and subsequently to 100 J/cm^2^ of laser irradiation. A 6.86 log_10_ CFU/mL and 5.4 log_10_ CFU/mL reduction was found in the monomicrobial biofilm of *S. aureus* and *P. aeruginosa*, respectively, whereas 5.31 log_10_ CFU/mL reductions were observed in the polymicrobial biofilms of both the species (Fig. 4). The bacterial load reduction in the polymicrobial biofilms of *S. aureus* and *P. aeruginosa* was found to be lower than monomicrobial biofilms of both the species after nano-photodynamic therapy (Supplementary Fig. S1).

### Generation of reactive oxygen species and singlet oxygen quantification

**Figure 5:**
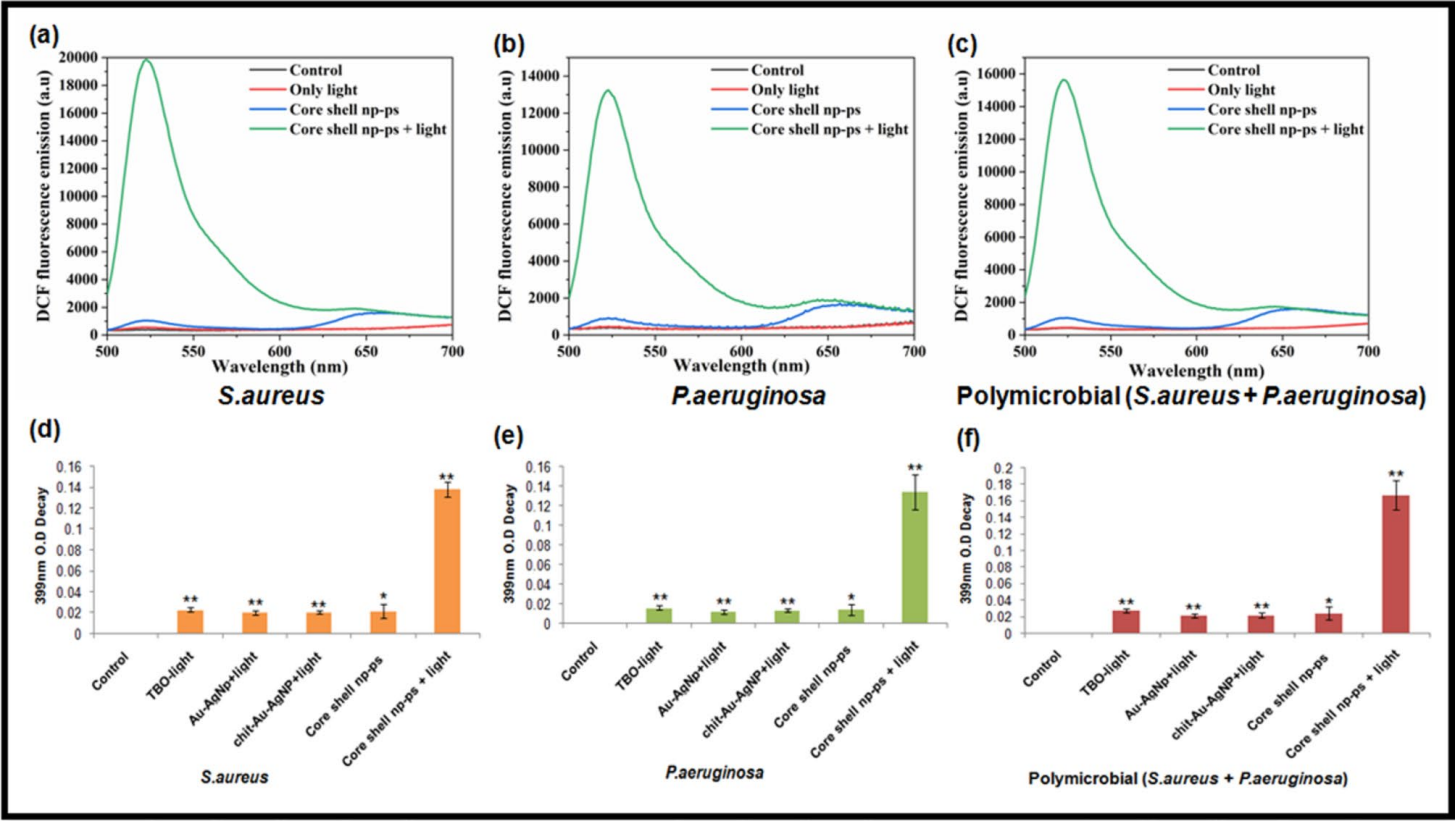
Detection of total reactive oxygen species (**a**)–(**c**) and quantification of singlet oxygen (**d**)–(**f**) in monomicrobial *S. aureus*, monomicrobial *P. aeruginosa* and polymicrobial *S. aureus* + *P. aeruginosa* biofilms in control group, TBO–chit–Au–AgNPs treated group and TBO–chit–Au–AgNPs + laser treated group. 630 nm laser (100 J/cm^2^, 12 min and 50 s) were used in the corresponding laser group. The data represents an average of triplicate experiments ± SD (*p*-value *p < 0.01, **p < 0.001, ***p < 0.0001, ns = not significant).

Our data showed increase production of intracellular ROS in TBO–chit–Au–AgNPs mediated photodynamic therapy treated group as compared to TBO–chit–Au–AgNPs alone, both in monomicrobial as well as in polymicrobial biofilms. Whereas, no significant ROS production was seen in the group treated with only light. Therefore, we have not selected only light treated group for further studies. Thus, we conclude that the potentiation of killing or enhanced antibacterial activity is dependent upon the generation of ROS (Fig. 5a–c). However, the photoinactivation was found to be more pronounced in monomicrobial biofilm than polymicrobial biofilms of both the species.

Furthermore, to confirm the type of phototoxicity, we have measured the degradation rate of AMDA. Since, the amount of ROS produced is directly proportional to the bacterial cell death; our data revealed enhanced production of ^1^O_2_ in TBO–chit–Au–AgNPs mediated PDT treated group as compared to control and only TBO–chit–Au–AgNPs treated groups (Fig. 5d–f). This confirmed that type II photochemistry is the major photochemical reaction involved in TBO–chit–Au–AgNPs mediated photodynamic therapy on monomicrobial and polymicrobial biofilms of *S. aureus* and *P. aeruginosa*.

### Anti-biofilm effect of TBO–chit–Au–AgNPs mediated photodynamic therapy

**Figure 6:**
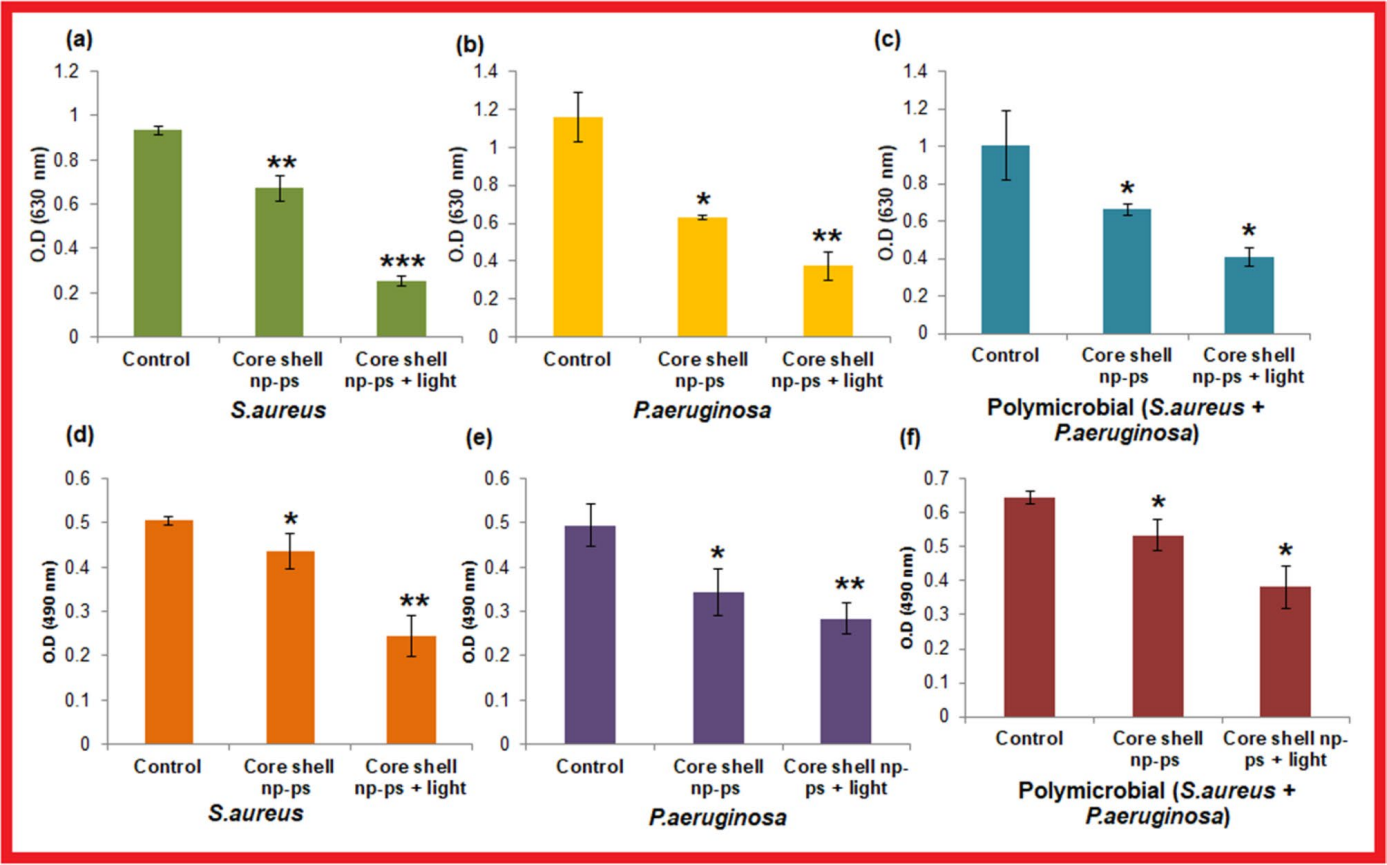
Antibiofilm forming ability of TBO–chit–Au–AgNPs mediated photodynamic therapy: (**a**)–(**c**) represents inhibitory effect of TBO–chit–Au–AgNPs mediated PDT on monomicrobial *S. aureus*, monomicrobial *P. aeruginosa* and polymicrobial *S. aureus* + *P. aeruginosa* biofilms, respectively as quantified by Crystal violet assay. Absorbance was measured at 630 nm. Figure (**d**)–(**f**) showed effect of TBO–chit–Au–AgNPs mediated PDT on extracellular polysaccharide substance (EPS) reduction as quantified by Congo-red assay. Absorbance was measured at 490 nm. The data represent an average of triplicate experiments ± SD. (*p*-value *p < 0.01, **p < 0.001, ***p < 0.0001, ns = not significant).

The result of crystal violet assay showed 28.02% and 34.04% reduction in the monomicrobial *S. arureus* and *P. aeruginosa* biofilm formation, respectively, after treatment with, exclusively TBO–chit–Au–AgNPs, whereas 45.89% of polymicrobial biofilms were found to be reduced. Moreover, significant decrease in microbial biomasses was found in TBO–chit–Au–AgNPs mediated photodynamic therapy treated group as compared to control and exclusively TBO–chit–Au–AgNPs treated groups. Our data showed 72.82% and 59.15% reduction in the monomicrobial biofilm of *S. aureus* and *P. aeruginosa,* respectively, while 67.69% reduction was found in the polymicrobial biofilms after treatment with TBO–chit–Au–AgNPs and subsequently to 100 J/cm^2^ of laser irradiation (Fig. 6a–c; Tables S1, S2). Besides this, EPS production was reduced by 13.65% and 30.71% in monomicrobial biofilm of *S. aureus* and *P. aeruginosa* whereas 17.16% in polymicrobial biofilms after being treated with exclusively TBO–chit–Au–AgNPs. While, 51.55% and 42.71% EPS reduction was achieved in monomicrobial biofilm of *S. aureus* and *P. aeruginosa,* respectively, after being photoinactivated by TBO–chit–Au–AgNPs. However, 40.9% reduction was seen in polymicrobial biofilms as shown in Fig. 6d–f.

### Visualization of biofilms architect after TBO–chit–Au–AgNPs mediated photodynamic therapy

**Figure 7:**
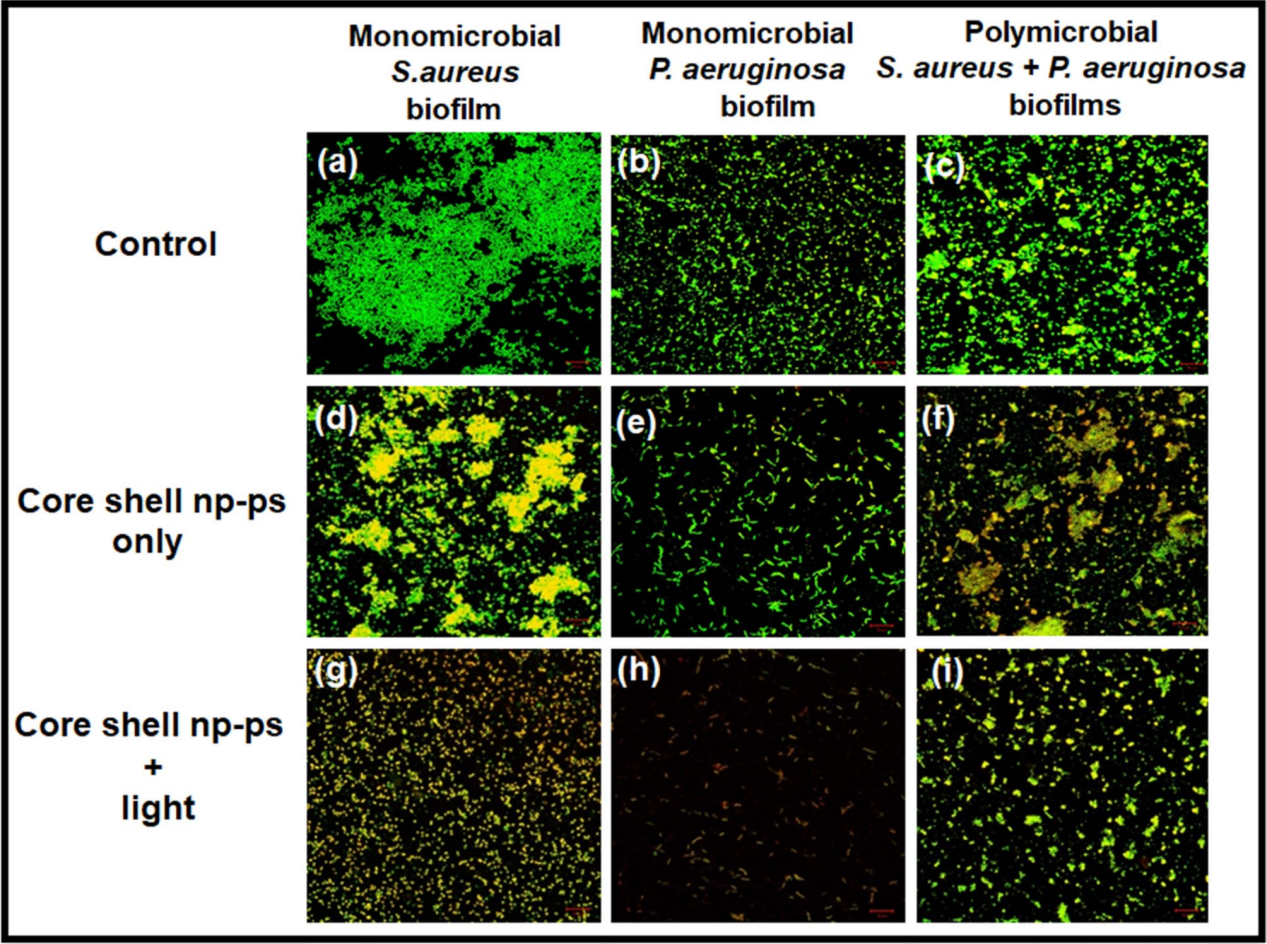
Fluorescence-based live/dead analysis of monomicrobial *S. aureus*, monomicrobial *P. aeruginosa* and polymicrobial *S. aureus* + *P. aeruginosa* biofilms: Representative fluorescence images of SYTO 9 (live, green) and PI (dead, red) stained bacteria in the groups of control (**a**)–(**c**), TBO–chit–Au–AgNPstreated (**d**)–(**f**) and TBO–chit–Au–AgNPs + laser treated (**g**)–(**i**). 630 nm laser (100 J/cm^2^, 12 min and 50 s) were used in the corresponding laser group. Scale bar = 10 μm.

**Figure 8:**
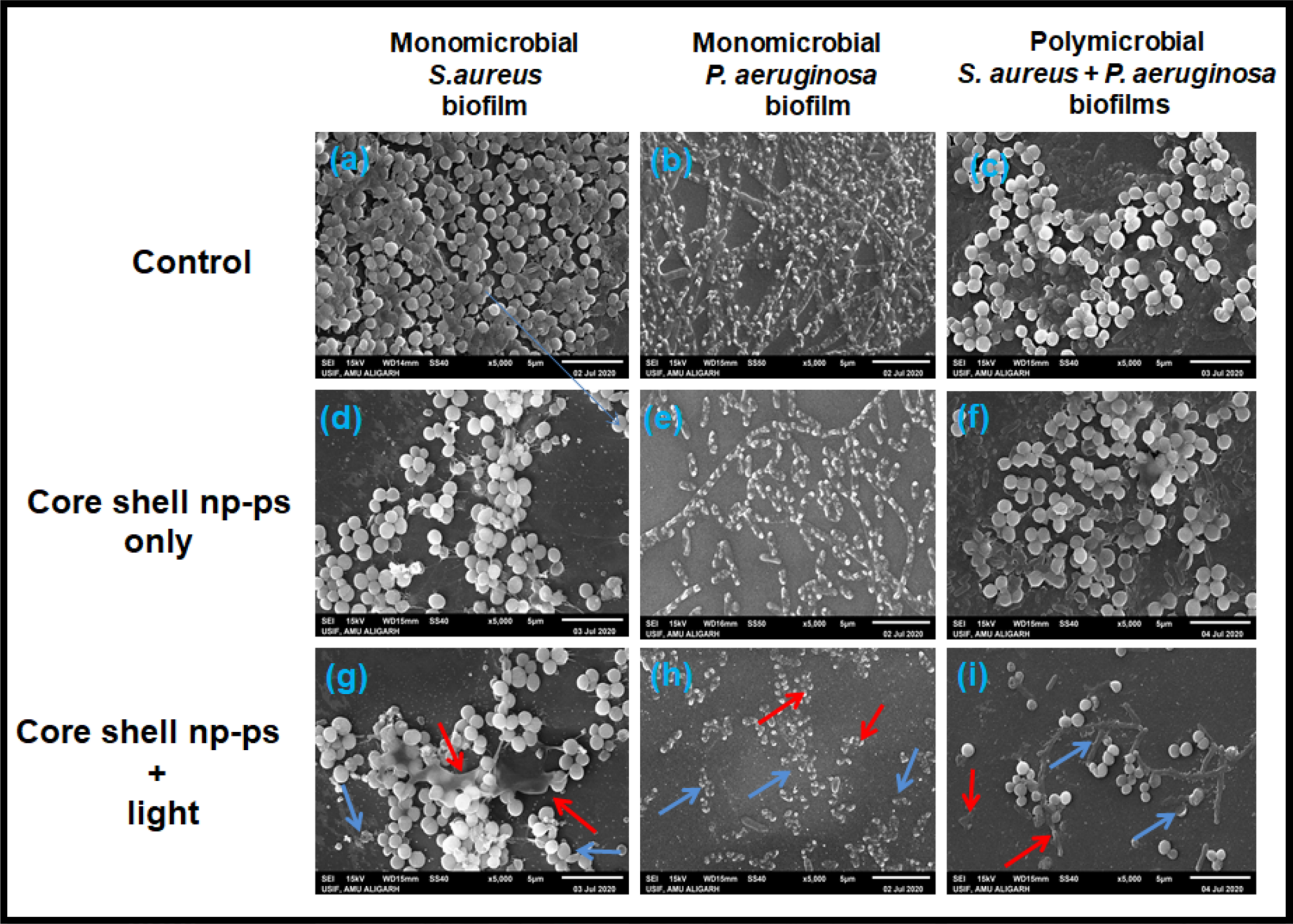
Scanning electron microscopy (SEM) images of monomicrobial *S.aureus*, monomicrobial *P. aeruginosa* and polymicrobial *S. aureus* + *P. aeruginosa* biofims of control (**a**)–(**c**), TBO–chit–Au–AgNPs treated (**d**)–(**f**) and TBO–chit–Au–AgNPs + laser treated (**g**)–(**i**). 630 nm laser (100 J/cm^2^, 12 min and 50 s) were used in the corresponding laser group. Red arrow indicates bursting and release of cellular constituents while blue arrow indicates complete rupturing of the cells.

Confocal and scanning electron microscopy was performed to examine the morphological changes in bacteria after TBO–chit–Au–AgNPs mediated photodynamic therapy, and to elucidate the underlying anti-bacterial mechanisms. CLSM micrographs illustrate greater disruption of polymicrobial biofilms of *S. aureus* and *P. aeruginosa* in TBO–chit–Au–AgNPs mediated photodynamic therapy treated group (Fig. 7i) as compared to control (Fig. 7c) and exclusively TBO–chit–Au–AgNPs (Fig. 7f). Moreover, the monomicrobial biofilms of *S. aureus* and *P. aeruginosa* was more severely disrupted, almost all the bacterial cells in the biofilms were found dead in TBO–chit–Au–AgNPs mediated PDT group (Fig. 7g, h), as compared to control (Fig. 7a, b) and exclusively TBO–chit–Au–AgNPs treated group (Fig. 7d, e). Thereby, demonstrating strong antibiofilm action of TBO–chit–Au–AgNPs mediated photodynamic therapy. Furthermore, the thickness of the biofilm was also recorded (Supplementary Fig. S2). TBO–chit–Au–AgNPs treatment reduced the thickness of the biofilm to approximately 4 µm and 3 µm in monomicrobial biofilm of *S. aureus* and *P. aeruginosa,* respectively*,* as compared to control (6 µm and 4 µm, respectively). However, the thickness was found to be 2.5 µm and 2 µm, respectively in TBO–chit–Au–AgNPs mediated PDT treated group, suggesting prevalence of dead cells throughout the biofilm. Likewise, polymicrobial biofilm of *S. aureus* and *P. aeruginosa* treated with TBO–chit–Au–AgNPs mediated PDT decreases the thickness of the biofilm to approximately 3 µm as compared to control (8 µm) and TBO–chit–Au–AgNPs (6 µm).This observation was further supported by the scanning electron microscopy. The control groups of monomicrobial as well as polymicrobial *S. aureus* and *P. aeruginosa* biofilms displayed highly dense and compact microbial cells (Fig. 8a–c). While, after treatment with TBO–chit–Au–AgNPs followed by exposure to 100 J/cm^2^ of laser light, the density of the microbial cells in the monomicrobial as well as in the polymicrobial biofilms of both the species decreases significantly (Fig. 8g–i). Less reduction was observed in only TBO–chit–Au–AgNPs treated groups (Fig. 8d–f). Furthermore, TBO–chit–Au–AgNPs mediated PDT treated groups showed detrimental effects on cell wall with significant dispersion of the cells leading to leakage of the cellular content, thus obliterating the structural integrity of the biofilm.

### In vivo efficacy of TBO–chit–Au–AgNPs mediated photodynamic therapy in the treatment of Diabetic Foot Ulcer

**Figure 9:**
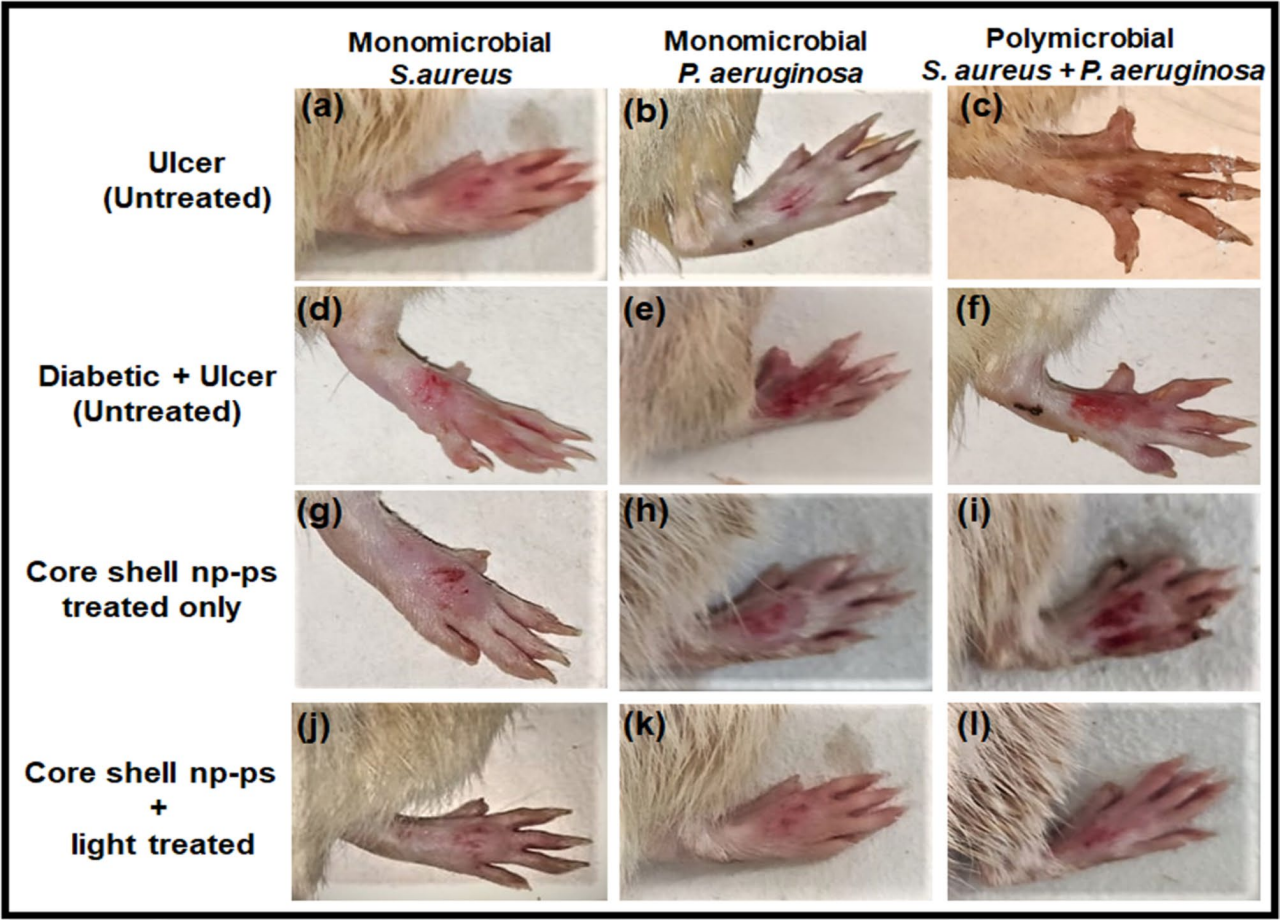
Photographic image of wistar rats: Untreated foot ulcer (**a**)–(**c**), untreated foot ulcer with diabetes (**d**)–(**f**), diabetic foot ulcer after applying TBO–chit–Au–AgNPs (**g**)–(**i**) and diabetic foot ulcer after treatment with TBO–chit–Au–AgNPs mediated PDT.

**Figure 10:**
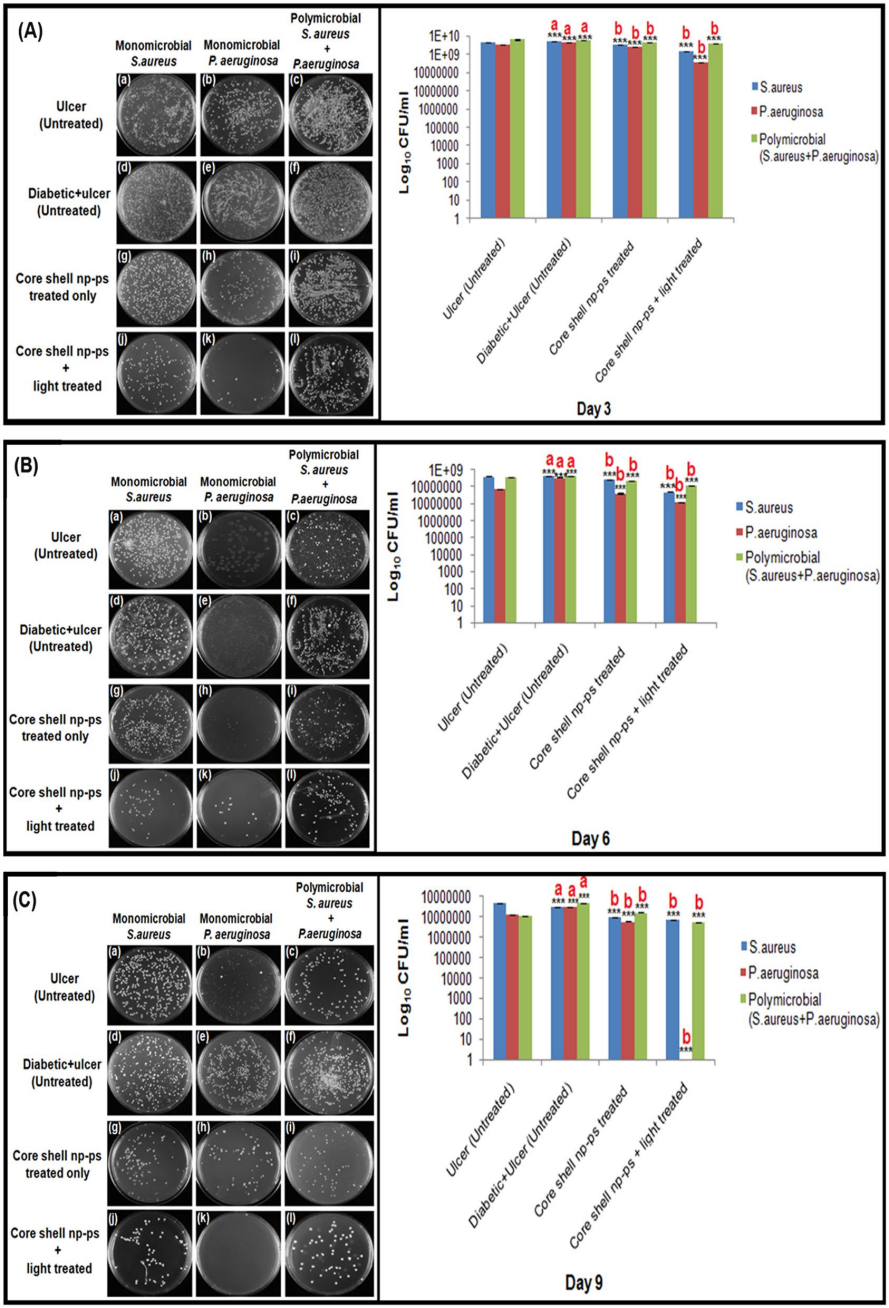
In vivo antibacterial effect of TBO–chit–Au–AgNPs mediated photodynamic therapy in DFU rats: (**A**) CFU/ml at day 3 post ulceration, (**B**) CFU/ml at day 6 post ulceration and treatment, (**C**) CFU/mL at day 9 post ulceration and treatment. Data are presented as mean ± SD (n = 4) and normalized to that of untreated control. Three replicates were performed for each experiment. Statistical significance was determined using one-way analysis of variance (*p*-value *p < 0.01, **p < 0.001, ***p < 0.0001, ns = not significant). a—compared with untreated ulcer; b—compared with untreated diabetic foot ulcer.

**Figure 11:**
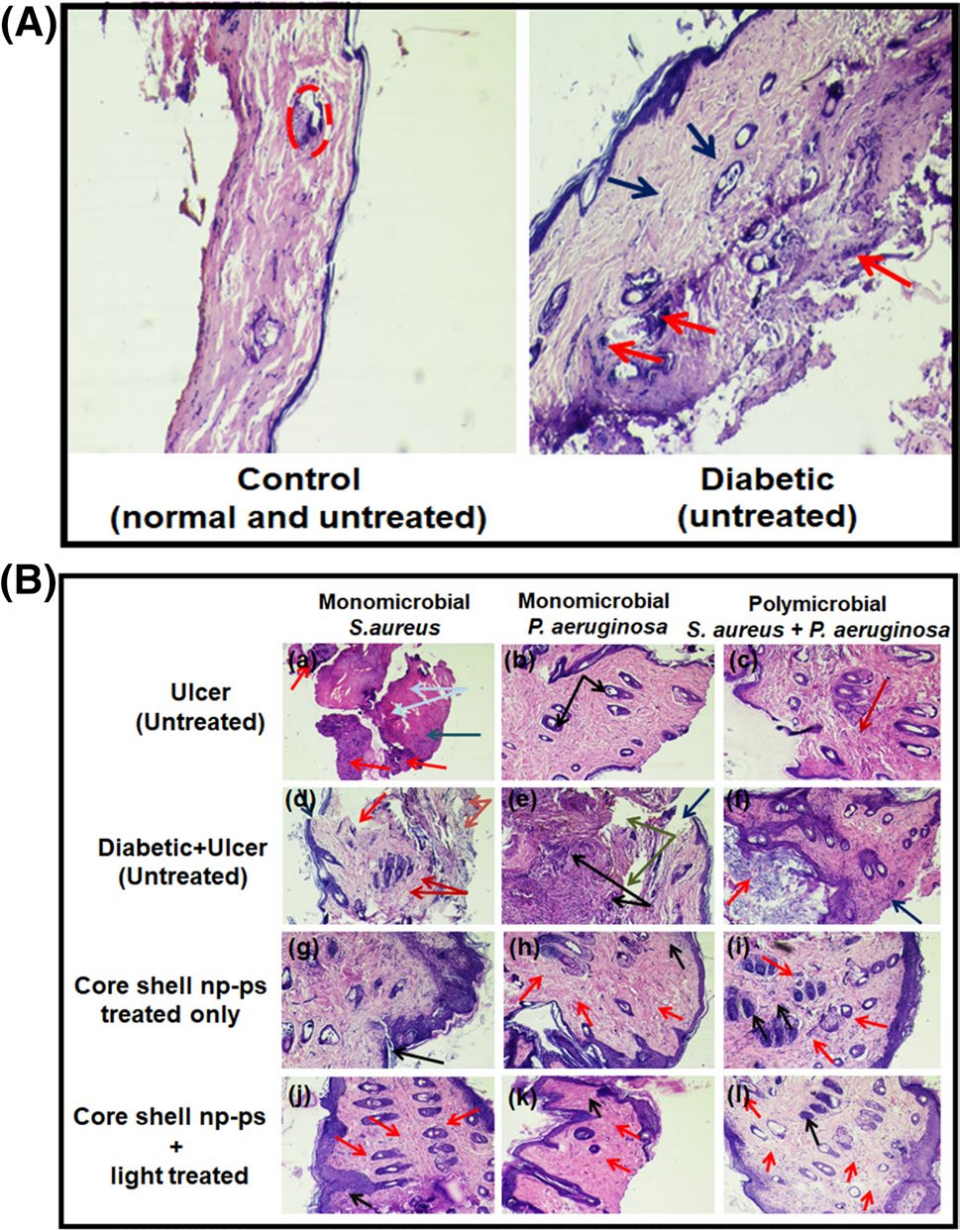
Hematoxylin and Eosin (H& E) (×10X) staining to examine response to microorganism of: (**A**) Control and diabetic rats, (**B**) Ulcerated rats (untreated): figure (**a**)–(**c**), DFU rats (untreated): figure (**d**)–(**f**), DFU rats treated with TBO–chit–Au–AgNPs: figure (**g**)–(**i**) and DFU rats treated with TBO–chit–Au–AgNPs + laser: figure (**j**)–(**l**). Red dashed line indicates sebaceous gland in Fig. 10(**A**) Control group, blue arrow indicates thin collagen fibres while red arrow indicates the locations of neutrophils in diabetic group. In Fig. 10(**B**), (a) blue arrow indicates necrosis, grey arrow indicates fibroblasts cells while red arrow indicates dense focal neutrophilic infilterate, (b) black arrow indicates hair follicles, (c) red arrow indicates mild fibrosis, (d) blue arrow indicates ulceration, red arrow indicates collagen fibres, brown arrow indicates thin capillaries while dark brown arrow indicates mild fibrosis, (e) black arrow indicates capillaries, grey arrow indicates collagen fibres while blue arrow indicates ulcer, (f) red arrow indicates squamous epithelium with marked keratosis whereas blue arrow indicates ulcer, (g) black arrow indicates healing ulcer, (h)-(l) red arrow indicates capillaries while black arrow indicates neutrophils.

The efficacy of TBO–chit–Au–AgNPs mediated photodynamic therapy in healing of diabetic foot ulcer in male wistar rats was checked. Streptozotocin (STZ)-induced type 2 DM rats with blood glucose level higher than 300 mg/dl were developed and used for further study (Table 2). Change in weight of the rats during the course of the experiment were also monitored (Table S3). Our result showed that daily topical exposure of TBO–chit–Au–AgNPs followed by light irradiation led to the marked reduction of *S. aureus* and *P. aeruginosa* colonization in diabetic foot ulcer rats within 7 days, starting from the day 3 post infection (Figs. 9, 10A–C, Table S4). Furthermore, histopathological analysis of untreated monomicrobial and polymicrobial DFU (Fig. 11B-d–f) revealed dense population of inflammatory cells with focal neutrophilic infiltrate attached to the stratified squamous epithelium as compared to control, diabetic (Fig. 11A) and ulcerated groups (Fig. 11B-a–c). In addition, the ulcerated squamous epithelium in untreated DFU rats showed decrease collagenization with few basal thin capillaries and mild fibrosis. This confirms the incidence of an ongoing infection. However, the ulcer sites of the TBO–chit–Au–AgNPs treated group showed acanthotic (thickened) squamous epithelium with moderate fibrosis. Besides this, restorative ulcer was observed with focal moderately thick collagen fibers and few compressed capillaries (Fig. 11B-g–i). In comparison, TBO–chit–Au–AgNPs mediated photodynamic therapy treated DFU showed intact stratified squamous epithelium with marked neo-angiogenesis and collagenization (Fig. 11B-j–l).

**Table 2:** Experimental induction of type-2 diabetes by streptozotocin (STZ) injection.

S. no.	Groups	Blood sugar concentration (mg/dL) in overnight fasting rats before the injection of Streptozotocin (STZ)	Blood sugar concentration (mg/dL) after the injection of Streptozotocin (STZ)
1	Control (normal rats)	i. 102 ii. 92 iii. 87 iv. 108	–
2	Diabetic without ulcer (untreated rats)	i. 98 ii. 90 iii. 89 iv. 99	i’. 368 ii’. 239 iii’. 366 iv’. 261
3	*Pseudomonas aeruginosa* Diabetic with ulcer (untreated rats)	i. 110 ii. 102 iii. 94 iv. 87	i’. 325 ii’. 338 iii’. 302 iv’. 319
4	*Pseudomonas aeruginosa* Diabetic with ulcer (TBO–chit–Au–AgNPs treated rats)	i. 95 ii. 130 iii. 94 iv. 102	i’. 380 ii’. 397 iii’. 394 iv’. 382
5	*Pseudomonas aeruginosa* Diabetic with ulcer (TBO–chit–Au–AgNPs + light treated rats)	i. 108 ii. 100 iii. 80 iv. 98	i’. 376 ii’. 265 iii’. 277 iv’. 264
6	*Staphylococcus aureus* Diabetic with ulcer (untreated rats)	i. 138 ii. 112 iii. 98 iv. 104	i’. 309 ii’. 386 iii’. 287 iv’. 312
7	*Staphylococcus aureus* Diabetic with ulcer (TBO–chit–Au–AgNPs treated rats)	i. 139 ii. 87 iii. 94 iv. 93	i’. 299 ii’. 310 iii’. 264 iv’. 317
8	*Staphylococcus aureus* Diabetic with ulcer (TBO–chit–Au–AgNPs + light treated rats)	i. 130 ii. 123 iii. 117 iv. 103	i’. 385 ii’. 305 iii’. 377 iv’. 338
9	Polymicrobial (*Staphylococcus aureus* + *Pseudomonas aeruginosa*) Diabetic with ulcer (untreated rats)	i. 104 ii. 110 iii. 96 iv. 117	i’. 291 ii’. 271 iii’. 359 iv’. 331
10	Polymicrobial (*Staphylococcus aureus* + *Pseudomonas aeruginosa*) Diabetic with ulcer (TBO–chit–Au–AgNPs treated rats)	i. 109 ii. 106 iii. 103 iv. 122	i’. 362 ii’. 358 iii’. 376 iv’. 321
11	Polymicrobial (*Staphylococcus aureus* + *Pseudomonas aeruginosa*) Diabetic with ulcer (TBO–chit–Au–AgNPs + light treated rats)	i. 106 ii. 114 iii. 73 iv. 111	i’. 319 ii’. 375 iii’. 332 iv’. 345

### Effect of TBO–chit–Au–AgNPs mediated photodynamic therapy on growth factors and inflammatory cytokines in diabetic foot ulcer development and healing

The expression levels of growth factors and cytokines in control (normal) rats were compared with diabetic and non-diabetic rats with healed (treated) and unhealed (untreated) ulcers.

**Figure 12:**
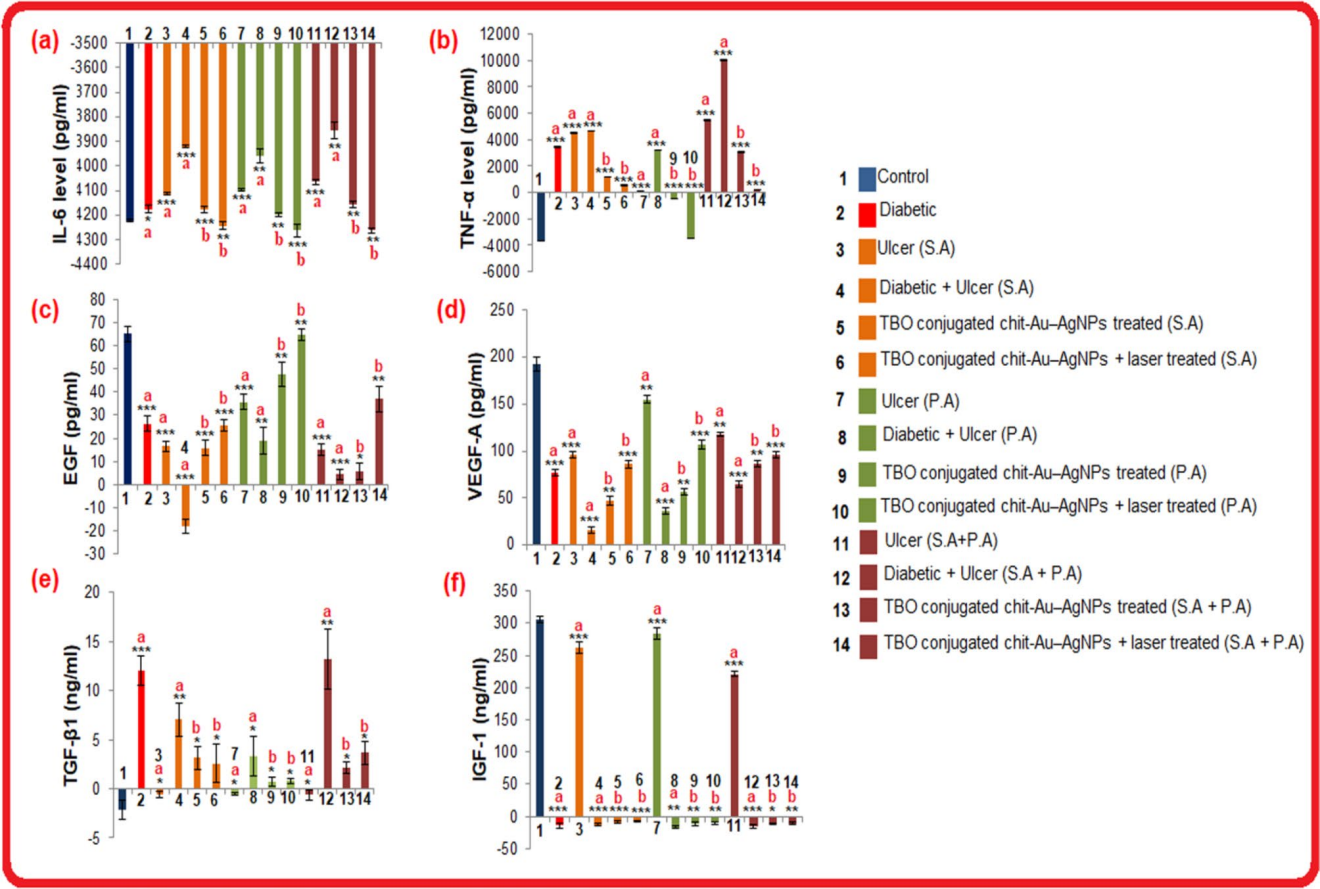
Effect of TBO–chit–Au–AgNPs mediated photodynamic therapy on pro-inflammatory cytokines and growth factors levels in Control rats, STZ induced diabetic rats (untreated), Ulcerated rats (untreated), DFU rats (untreated), DFU rats treated with TBO–chit–Au–AgNPs, DFU rats treated with TBO–chit–Au–AgNPs + laser: (**a**) IL-6 level, (**b**) TNF-α level, (**c**) EGF level, (**d**) VEGF-A level, (**e**) TGF-β1 level and (**f**) IGF-1 level. Each bar represents mean ± SD of 4 rats. Significance at: p-value *p < 0.01, **p < 0.001, ***p < 0.000, ns = not significant. a—compared with control or normal rats; b—compared with untreated diabetic foot ulcer rats.

We have found that failure of an ulcer to heal in diabetic rats was linked with an increased level of pro-inflammatory cytokines, such as IL-6 and TNF-α. Furthermore, the expression levels of pro-inflammatory cytokines were significantly higher in DFU rats with polymicrobial infections as compared to DFU rats with monomicrobial infection. However, decreased levels of these cytokines were found in TBO–chit–Au–AgNPs mediated photodynamic therapy treated groups as compared to control, untreated and only TBO–chit–Au–AgNPs treated groups, implying restoration of immunosuppression (Fig. 12-a, b). In addition, our data demonstrated significantly higher levels of EGF and VEGF in all those groups of rats whose ulcers healed as compared to those groups whose ulcers did not healed (Fig. 12-c, d). However, their expression level was found to be lower in DFU rats with polymicrobial infections than that of monomicrobial infection, as the severity of infection was found to be more in polymicrobial state of infection. Besides this, elevated level of TGF-β-1 was found in all diabetic groups (with and without foot ulcer) (Fig. 12e). Moreover, we have found expression levels of TGF-β-1 were significantly higher in diabetic rats with polymicrobial infections as compared to monomicrobial infection. In addition, IGF-1 level was found to be lower in diabetic rats as compared to non-diabetic and control rats. However, considerable decrease was found in DFU rats with polymicrobial infections (Fig. 12f). Hence, the data suggest that TBO–chit–Au–AgNPs mediated PDT promotes healing in DFU rats through significantly reducing cytokine production while elevating EGF and VEGF levels by regulating the expression of TGF-β-1 and IGF-1.

## Discussion

Type 2 diabetes mellitus (DM) has become an utmost health concern which comprised of 90–95% diabetes among worldwide population^1^. It has also been reported that up to one-third of people with diabetes may develop foot ulcer during their lifetime and over 50% of these ulcers become infected. This challenge is further aggravated by the emergence of multidrug-resistance (MDR) strains induced diabetic foot infections (DFIs) which leads to increasing morbidity and mortality, and risk of lower extremity amputation (LEA) which causes low quality of life^3–6^. Majority of these infections are now being caused by *Pseudomonas aeruginosa*, *Staphylococcus aureus*, *Escherichia coli*, *Enterococcus* spp., *Streptococcus* spp., *Proteus mirabilis* and anaerobes^11^.

*S. aureus* and *P. aeruginosa* are two versatile bacterial pathogens that are frequently found together in chronic wound infections^12,50^. Polymicrobial (mixed microbial culture) *S. aureus* and *P. aeruginosa* infections are more virulent and/or result in worse outcomes than the single infections caused by either species^15^, leading to the formation of more resistant biofilms, which are difficult to be eradicated. For instance, biofilm-forming MDR strains are far more impervious to antimicrobials than organisms in suspension^7–9^. In this regard, nanoparticle mediated photodynamic therapy emerged as an ideal candidate to meet such requirements^51,52^.

Recently, chitosan coated gold–silver core–shell nanoparticles (chit–Au–AgNPs) was developed and labeled with para-mercaptobenzoic acid (4MBA) to demonstrate their ability to perform as SERS nanotags inside of human ovarian adenocarcinoma cells (NIH: OVCAR-3) under multiple excitation wavelengths^24^. Here, chit–Au–AgNPs were used for the first time in association with TBO.

Earlier studies have shown that photosensitizer activity is significantly improved when combine with nanoparticles^49^. Our study was planned to investigate the effectiveness of novel toluidine blue conjugated gold–silver core–shell nanoparticles (TBO–chit–Au–AgNPs) mediated photodynamic therapy and demonstrate their use as a nontoxic antibacterial therapy to combat diabetic foot ulcer (DFU) caused by multi-drug resistant strains both in monomicrobial and polymicrobial state of infection.

The synthesized TBO–chit–Au–AgNPs were characterized by UV-spectroscopy, TEM, scanning electron microscopy (SEM) with energy dispersive X-ray spectrometry (EDS), DLS and Zeta potential. The UV–visible spectrum of the synthesized chit–AuNPs (Fig. 2A(f)-blue spectrum) exhibits a well-defined SPR (surface plasmon resonance) band centered at 530 nm. For instance, occurrence of a new band takes place at 405 nm (Fig. 2A(f)-red spectrum) upon first addition of AgNO_3_ aliquots. This spectral profile has already been reported in previous studies and it confirms the successful synthesis of a thin silver layer on the surface of the gold core^53^. The morphological progression of core–shell nanoparticles (chit–Au–AgNPs) was further confirmed by TEM. The dark gold core and the brighter silver shell as observed in Fig. 2A(b–d), was clearly distinguishable because of higher electronic density of gold than silver. Furthermore, Fig. 2A(e) showed the presence of chitosan layer as a faint shadow which encapsulates the Au–AgNPs after their formation. This foremost result demonstrated not only the effective synthesis of Au–AgNPs, but also their steadiness and bio-compatibilization, which are required essentially for the use of this nanoconjugate in biological systems. We next performed elemental mapping to verify the presence of gold and silver using scanning electron microscopy (SEM) with energy dispersive X-ray spectrometry (EDS). The result confirms the formation of chit–Au–Ag nanocomposites with no additional impurities as detected in the EDS (Fig. 2B-a–c). Changes in the polydispersity index (PDI) were also measured over this time. The PDI of chit-AuNPs, chit–Au–AgNPs and TBO–chit–Au–AgNPs remained low, indicating that there is least or no aggregation of particles (Table 1). We have also measured the surface zeta potential to further confirm about the stability of nanoparticles. Our data demonstrated strong positive zeta potential of chit–Au–AgNPs (+41.2 mV) even after the addition of TBO, suggesting the stability and biocompatibility of synthesized core–shell nanoparticles (chit–Au–AgNPs) with TBO.

We next checked the cytotoxicity of TBO–chit–Au–AgNPsin the presence as well as in the absence of laser irradiation. As presented in Fig. 3, the synthesized TBO-chit–Au–AgNPs in the presence of laser irradiation did not exhibit any obvious cytotoxic effects at our tested concentration (0.5 mM). This could be attributed mainly to the biological and chemical properties of chitosan, as well as low toxic effect of TBO on fibroblast cells^54,55^. Furthermore, the wavelength of red laser (630 nm) lies in the visible spectrum. Hence, the concentration of the TBO–chit–Au–AgNPs and exposure time of red laser light used in this study was found to be non-toxic.

One of the main factors responsible for the resistance of *S. aureus* and *P. aeruginosa* to both antibiotics and immune cells is their propensity to form biofilms. We therefore assessed the antibacterial activity of TBO–chit–Au–AgNPs in the presence as well as in the absence of laser irradiation using colony formation assay against polymicrobial biofilms and compared with that of monomicrobial biofilms. A substantial reduction in bacterial load was achieved when monomicrobial and polymicrobial biofilms of *S. aureus* and *P. aeruginosa* treated with TBO–chit–Au–AgNPs and subsequently to 100 J/cm^2^ of laser irradiation (Fig. 4). The bacterial load reduction in the polymicrobial biofilms of *S. aureus* and *P. aeruginosa* was found to be lower than monomicrobial biofilms of both the species after nano-photodynamic therapy (Supplementary Fig. S1). The results from the present study illustrated synergistic interaction between *S. aureus* and *P. aeruginosa* which is in concordance with previous studies^13^. Moreover, we have found Gram-positive, *S. aureus* as more sensitive towards nanoparticle mediated photodynamic therapy as compared to Gram-negative, *P. aeruginosa.* Since, it has been reported in the literature that teichoic acid found within the cell wall of Gram-positive bacteria is the main binding site of some small molecules and nanoparticles^56^. The improved susceptibility of *S. aureus*, despite of thick peptidoglycan layer may be attributed to the interaction between positively charged TBO–chit–Au–AgNPs and the anionic teichoic acid which leads to the cleavage of the peptidoglycan layer and pore formation in the membrane.

In order to illustrate the above outcomes, we further analyzed the mechanism behind the antibacterial activity of TBO–chit–Au–AgNPs mediated photodynamic therapy using DCFH-DA and quantified the production of total reactive oxygen species (ROS) under laser irradiation. Our data showed increase production of intracellular ROS in TBO–chit–Au–AgNPs mediated photodynamic therapy treated group as compared to TBO–chit–Au–AgNPs alone, both in monomicrobial as well as in polymicrobial biofilms. However, the photoinactivation was found to be more pronounced in monomicrobial biofilm than polymicrobial biofilms of both the species. Thus, we conclude that the potentiation of killing or enhanced antibacterial activity is dependent upon the generation of ROS (Fig. 5a–c). Additionally, bacterial cells have sufficient amount of scavengers such as catalase, peroxidase and superoxide dismutase to thwart the free radical mediated bactericidal activity, however they have no remedy against the singlet oxygen molecule, as a result, ^1^O_2_ leads to maximum cell damage^57^. Therefore, to confirm the type of phototoxicity, we have used AMDA. Our data revealed enhanced production of ^1^O_2_ in TBO–chit–Au–AgNPs mediated PDT treated group as compared to control and only TBO–chit–Au–AgNPs treated groups (Fig. 5d–f).

Furthermore, the ability of bacterial adherence and exopolysaccharide production which are significantly important for the formation of biofilm architecture, were analyzed by crystal violet (CV) and congo red (CR)-binding assays, respectively. Our data revealed polymicrobial biofilm was more impervious and difficult to eradicate than monomicrobial biofilm^58^ (Fig. 6). This may be due to the presence of more than one type of EPS formed by the bacteria which resulted into more viscous matrix^59^.

We next perform confocal and scanning electron microscopy to further examine the morphological changes in bacteria after TBO–chit–Au–AgNPs mediated photodynamic therapy, and to elucidate the underlying anti-bacterial mechanisms. CLSM micrographs illustrate disruption of polymicrobial biofilms of *S. aureus* and *P. aeruginosa* in TBO–chit–Au–AgNPs mediated photodynamic therapy treated group as compared to control and exclusively TBO–chit–Au–AgNPs treated groups. Moreover, the monomicrobial biofilms of *S. aureus* and *P. aeruginosa* was found to be more severely disrupted, almost all the bacterial cells in the biofilms were found dead in TBO–chit–Au–AgNPs mediated PDT group, as compared to control and exclusively TBO–chit–Au–AgNPs treated group (Fig. 7). Thereby, demonstrating strong antibiofilm action of TBO–chit–Au–AgNPs mediated photodynamic therapy (Supplementary Fig. S2). This observation was further supported by the scanning electron microscopy. The control groups of monomicrobial as well as polymicrobial *S. aureus* and *P. aeruginosa* biofilms displayed highly dense and compact microbial cells. While, after treatment with TBO–chit–Au–AgNPs followed by exposure to 100 J/cm^2^ of laser light, the density of the microbial cells in the monomicrobial as well as in the polymicrobial biofilms of both the species decreases significantly (Fig. 8). Furthermore, TBO–chit–Au–AgNPs mediated photodynamic therapy treated groups showed detrimental effects on cell wall with significant dispersion of the cells leading to leakage of the cellular content, thus obliterating the structural integrity of the biofilm. It has already been reported that Au–Ag nano-shells increases membrane permeability which in turns facilitate the intracellular transport of more AuAg nano-shells, primarily at elevated temperature upon laser irradiation which maximizes the antibacterial efficacy of AuAgNSs^19^.

Additionally, the efficacy of TBO–chit–Au–AgNPs mediated photodynamic therapy in healing of diabetic foot ulcer in male wistar rats was checked. Our result showed that daily topical exposure of TBO–chit–Au–AgNPs followed by light irradiation led to marked reduction of *S. aureus* and *P. aeruginosa* colonization in diabetic foot ulcer rats (Fig. 9). Previous study have shown that foot ulcers in the diabetic rats healed slower than those of the non-diabetic rats, however in the present study, TBO–chit–Au–AgNPs mediated PDT remarkably healed foot ulcer in diabetic rats within 7 days, after daily treatment (Fig. 10A–C). Such fast recovery might obviate the chances of having further bacterial infection and consequent mortality. Furthermore, the result of histopathological investigation of monomicrobial and polymicrobial DFU revealed that TBO–chit–Au–AgNPs mediated PDT promotes healing and reduces inflammation in DFU rats (Fig. 11A, B).

Foot ulcers in diabetic patients take a long time to heal due to a series of cellular and molecular mechanism employed in the process of healing, such as neuropathy, high probability of infection, non-physiological inflammatory response, lack of neoangiogenesis, oxidative stress, insufficient concentrations of growth factors, cellular abnormalities, etc.^1,60^. Therefore, in order to understand the pathogenesis and healing of DFU in monomicrobial and polymicrobial state of infection, we have performed serum analysis of several growth factors and pro-inflammatory cytokines. Our data showed decreased levels of IL-6 and TNF-α in TBO–chit–Au–AgNPs mediated photodynamic therapy treated groups as compared to control, untreated and only TBO–chit–Au–AgNPs treated groups, implying restoration of immunosuppression (Fig. 12-a, b). Furthermore, we have found higher levels of EGF and VEGF in all those groups of rats whose ulcers healed as compared to those groups whose ulcers did not healed (Fig. 12-c, d). This might be, because EGF promote ulcer healing by stimulating cell growth, proliferation and differentiation whereas, VEGF stimulates vasculogenesis and angiogenesis^61,62^. Besides this, elevated level of TGF-β-1 was found in all diabetic groups (with and without foot ulcer) (Fig. 12e). This is because TGF-β-1 is a systemic marker of type-2 diabetes and is positively associated with hyperglycemia. In addition, IGF-1 level was found to be lower in diabetic rats as compared to non-diabetic and control rats. However, considerable decrease was found in DFU rats with polymicrobial infections (Fig. 12f). IGF-1 contributes in cell granulation during wound healing; its expression decreases in diabetic patients which resulted into anomalies in cell granulation^63^. Thus, the findings of present study clearly indicate pathogenesis, healing of DFU and infection control.

## Materials and methods

### Compliance of ethical standards

This study was carried out in accordance with Institutional Animal Ethics Committee (IAEC) guidelines. The experiments on animals were approved by the “Jawaharlal Nehru Medical College, AMU, Institutional Animal Ethics Committee”, registration no. 401/GO/Re/S/2001/CPCSEA. All pertinent guidelines for the care and use of animals have taken in consideration. Moreover, the study reported is in accordance with ARRIVE guidelines.

### Materials

Chitosan, hydrogen tetrachloroaurate (III) trihydrate (HAuCl_4_·3H_2_O) and toluidine blue O (TBO) were procured from Sigma-Aldrich. Ascorbic acid, glacial acetic acid (99.8%), silver nitrate (AgNO_3_) and sodium hydroxide (NaOH) were purchased from Merck. All reagents used, were of analytical grade and the solutions were prepared using HPLC water. Prior to use, the glacial acetic acid was diluted to a 1% aqueous solution. Chitosan was dissolved in 1% acetic acid solution.

### Nanoparticles synthesis

Chitosan–coated gold–silver core–shell nanocomposites (chit–Au–AgNPs) were synthesized as described previously. At first, chitosan coated gold nanoparticles termed as ‘core’ was synthesized by mixing 15 mL of 10 × 10^−3^ M (10 mM) HAuCl_4_ and 90 mL chitosan solution under magnetic stirring at 50 °C. The appearance of a red color indicates the formation of spherical gold nanoparticles^64^.The next step involves 3 consecutive additions of 140 μL of 10 × 10^−1^ M of AgNO_3_ to a solution containing 70 mL of chit-AuNPs and 560 μL of 10 × 10^−1^ M of ascorbic acid, the latter used as reducing agent in this mixture. After each addition, the mixture was stirred at room temperature for 1 h. Thereafter, AgNO_3_ was added in a stepwise manner in an attempt to prevent the formation of free Ag nuclei^65^.

### Characterization

The stock solution of TBO was prepared in high performance liquid chromatography (HPLC) grade water. Later, the TBO solution was filtered-sterilized and stored at 4 °C in the dark until used. Further to confirm the conjugation of the TBO to chit–Au–AgNPs, a double-beam UV–visible spectrophotometer (PerkinElmer, Boston, MA, USA) was used. The baseline was fixed in the wavelength range 300–800 nm. We have added TBO (1 mg/mL) serially in 0.5 mM chit–Au–AgNPs and check the UV spectra for their interaction. Transmission electron microscope (TEM, JEM-2100F; Jeol, Tokyo, Japan) was employed to observe the size and morphology of the nanocomposites. The elemental constitution of the synthesized nanocomposites was analyzed using EDS spectroscopic techniques on INCAX-act, Model 51-ADD0076, Oxford instruments. Scanning electron microscopy (SEM) with energy dispersive X-ray spectrometry (EDS) were carried out to verify the presence of gold and silver. Hydrodynamic size and zeta potential of chit-AuNPs, chit–Au–AgNPs and TBO–chit–Au–AgNPs were recorded using Malvern Zetasizer (Nano ZS, Malvern, UK).

### Bacterial strains and culture condition

*S. aureus* and *P. aeruginosa* were used in this study. Brain heart infusion (BHI) broth (Himedia Labs, Mumbai, India) was used to culture bacteria at 37 °C for 24 h.

### Photosensitization and light source

Red diode laser (Model No-MRL-III-630; CNI, China) was used for photosensitization.

The effective radiant exposure of the light source was calculated using the following formula^66^:

$$ {\text{Energy fluency }} = {\text{ Power density }} \times {\text{ Time}} $$

where power density (PD) = P (mW)/Area (cm^2^), P represent the output power (100 mW) of laser and A indicates the area of irradiation. In this study, the laser treatment was carried out in a U-bottom microtiter plate. Thus, the samples acquire the shape of a hemisphere; hence the irradiated area was 2πr^2^, where r is the radius of the laser beam exposed, which is equal to 0.35 cm. The beam height from the base was 24.8 mm. Thus, the value of applied PD was 0.1300 W/cm^2^ and the energy fluency was set to 100 J/cm^2^ (12 min and 50 s) based on above mentioned formula.

### Estimation of in vitro antibacterial activity by colony forming assay

#### Monomicrobial biofilm

*Staphylococcus aureus* and *Pseudomonas aeruginosa* were grown overnight in BHI broth supplemented with 1% sucrose. Thereafter, the cell densities of the suspensions were adjusted to approximately 10^8^ CFU/mL using spectrophotometer (Optical Density; OD 0.8 at 600 nm). Hundred microliters of the diluted bacterial suspension were added into 96 well plates and incubated for 24 h at 37 °C. After incubation, the biofilms were gently washed with sterile phosphate-buffered saline (PBS) for two times. Subsequently, the preformed biofilm was incubated with TBO–chit–Au–AgNPs in the dark for 30 min and then irradiated with 100 J/cm^2^ (12 min and 50 s). Control (matured biofilm in phosphate buffer saline) and only TBO–chit–Au–AgNPs treated wells were not exposed to laser light. Finally, the resulting biofilm was then disrupted by vortexing followed by 10-fold serial dilution. 100 µL of the diluted suspension was spread onto BHI agar plate and then incubated at 37 °C for 24 h. A number of grown colonies were imaged and counted^67^.

#### Polymicrobial biofilms

To enumerate the CFUs in polymicrobial biofilms, *S. aureus* and *P. aeruginosa* were incubated overnight separately in BHI broth supplemented with 1% sucrose. Polymicrobial or mixed culture biofilms were formed by mixing 10^8^ CFU/mL cells from each bacterial suspension in fresh BHI broth and incubating for 24 h at 37 °C. After incubation, 10^8^ CFU/mL of mixed culture was taken and treated the way as defined above.

#### Total ROS detection inside the cells

2ʹ,7ʹ-Dichlorofluorescein-diacetate (DCFH-DA) was used to quantify endogenous ROS production in biofilms. Monomicrobial and polymicrobial *S. aureus* and *P. aeruginosa *biofilms were grown for 24 h as described above. After incubation, the cultures were centrifuged at 10,000 × *g* for 15min. Pellets were washed two times with PBS and finally re-suspended in it by adjusting the cell density to 10^8^ CFU/mL followed by incubation for 10 min with 10 μM DCFH-DA. At the end of incubation, the cells were treated with TBO–chit–Au–AgNPs and then irradiated with or without laser light. In, only light treated group, cells were exposed to 100 J/cm^2^ of laser light. Thereafter, the fluorescence intensity produced from DCFH-DA was estimated by excitation at 485 nm using slit width 1.5 nm^35^.

#### Evaluation of singlet oxygen in biofilms

9, 10-Anthracenediylbis (methylene) dimalonic acid (AMDA) was employed to quantify the ^1^O_2_ quantum yields of TBO–chit–Au–AgNPs with and without laser light treatment.

10 μM AMDA was added to the solutions containing monomicrobial and polymicrobial *S. aureus* and *P. aeruginosa* (10^8^ CFU/mL) biofilms in PBS followed by the treatment as explained above. The reduction in the 399-nm absorption peak of AMDA after treatment corresponds to the amount of singlet oxygen produced^68^.O.D in untreated group (control) was taken as 100%.

$$ {\text{Singlet oxygen generation }} = {\text{ OD of untreated group }}\left( {{\text{control}}} \right) \, - {\text{ OD of treated group}} $$

#### Quantification of biofilm formation by crystal violet assay

Crystal violet assay was performed to quantify the biomass of the monomicrobial and polymicrobial biofilms after treatment with TBO–chit–Au–AgNPs mediated photodynamic therapy. Preformed monomicrobial and polymicrobial biofilms of *S. aureus* and *P. aeruginosa* were grown for 24 h as described in colony forming assay. Prior to the treatment, the exhausted media was removed from each well. Thereafter, the biofilm was incubated with TBO–chit–Au–AgNPs for 30 min and then exposed to laser light (100 J/cm^2^). Subsequently, the control and treated (only TBO–chit–Au–AgNPs, TBO–chit–Au–AgNPs+ laser light treated) wells of microtiter plates were fixed with formalin (37%, diluted 1:10) and 2% sodium acetate. Biofilms in different groups were stained with 200 µL of crystal violet (0.1%, 15 min). These biofilms were washed twice with PBS; the unbound dye was removed with 100 μL of 95% ethanol. Thereafter, plates were shaken for 10 min to allow full release of the dye and the absorbance was recorded at 630 nm^35^.

### Congo red (CR)-binding assay

The Congo red (CR)-binding assay was conducted to estimate the production of exopolysaccharide (EPS), as reported earlier^69^. Same treatment was given on the preformed biofilm as described above. Thereafter, the exhausted media was removed and wells were washed with PBS twice. Hundred microliters of fresh medium and 50 μL of CR (0.5 mM) were added into each well containing control and treated samples. The mixture solution of fresh medium (100 μL) and CR (50 μL) was also used for blank measurements (blank CR). Subsequently, the microtiter plate was incubated for 2 h at 37 °C. After incubation, the content of each well was transferred to micro-centrifuge tubes followed by centrifugation at 10,000 × g for 5 min. The supernatants were collected and color change was measured at 490 nm^42^. The amount of EPS formed was measured using the following formula^70^:

$${\text{OD of bound CR (EPS produced)}}  =  {\text{OD of blank CR}} - {\text{OD of the supernatant}}.$$

### Live/dead staining by CLSM

Confocal laser scanning microscopy (CLSM) was performed to analyze the consequence of nano photodynamic therapy on monomicrobial and polymicrobial biofilm formation by *S. aureus* and *P. aeruginosa*. Biofilm was grown in covered glass bottom confocal dishes for 24 h at 37 °C. After incubation, unattached bacteria were gently washed away with sterile PBS. The adhered cells or the preformed biofilm was treated as describe above, while controls were left untreated. Then, the biofilm was stained with propidium iodide (PI) and syto9 followed by incubation at 37 °C for 1 h. Thereafter, confocal laser scanning microscope (Fluo View FV1000, TOKYO, JAPAN) was used to image the stained biofilms^35^.

### Structural imaging of bacteria in biofilms

Scanning electron microscopy (SEM) was executed to observe the morphology of *S. aureus* and *P. aeruginosa* monomicrobial as well as polymicrobial biofilms after treatment with TBO–chit–Au–AgNPs followed by exposure to laser light. The preformed biofilm was treated as describe above. After treatment, biofilms in each sample were fixed with 2% formaldehyde + 2.5% glutaraldehyde in PBS for 2 h at 4 °C. The fixed samples were serially dehydrated with different concentrations of ethanol (20%, 40%, 60%, 80%, and 100%). Finally, the samples were dried, mounted and sputter coated with gold-palladium for SEM analysis^35^.

### Cytotoxicity assay

A cytotoxicity assay was performed on L929 mouse fibroblast cells. The L929 mouse fibroblast cells were grown in Dulbecco’s modified Eagles medium (Sigma Aldrich, USA), supplemented with 10% fetal bovine serum (FBS) and 1% antibiotic i.e., penicillin-streptomycin (pen-strep) at 37 °C and 95% humidified incubator with 5% CO_2_. Sterile conditions were maintained all the times. Cells (∼10^5^cells*/*well) were seeded in 96-well plates, left overnight to adhere and then treated with various concentrations (0.25 mM, 0.5 mM and 1 mM) of TBO–chit–Au–AgNPs in the presence as well as in the absence of laser irradiation. After 24 h, cell viabilities were determined by methyl thiazolyl tetrazolium (MTT) assay. The medium was removed, and 100 μL of the mixture solution containing fresh medium and MTT (5 mg*/*mL) solution was added into each well, followed by incubation for 4 h at 37 °C. Subsequently, the formazan crystals formed by the reduction of MTT, were dissolved in DMSO and the absorbance was quantified by measuring its optical density at a wavelength of 570 nm using spectrophotometer^71^.

### In vivo study

A total of 56 adult male wistar rats, weighing, 250–300 g, were used in this study. The rats were equally divided into 6 main groups. Each group and sub-groups consisted of 4 rats each. Group 1 consisted of normal rats without type 2 diabetes mellitus (DM) and foot ulcer (Control); Group 2 contained rats with DM (Untreated); Group 3 consisted of rats with foot ulcer (Untreated); Group 4 contained rats with diabetic foot ulcer (Untreated); Group 5 consisted of diabetic foot ulcer rats treated with TBO–chit–Au–AgNPs while group 6 contained diabetic foot ulcer rats treated with TBO–chit–Au–AgNPs+ laser light (100 J/cm^2^).

Group 3 to group 6 is further sub-divided into 3 sub-groups, foot ulcerated with monoculture containing *S. aureus*, *P. aeruginosa,* and dual culture (*S. aureus* + *P. aeruginosa*), respectively. The animals were housed 4 per cage and kept on a 12-h light/dark cycle with access to food and water ad libitum.

### Experimental induction of diabetes

The animals were fasted overnight and diabetes was induced by a single intra-peritoneal injection of a freshly prepared solution of streptozotocin (40 mg/kg b. wt) in 0.1 M citrate buffer (pH 4.5)^1^. Control rats were injected with citrate buffer only. The rats were kept for 14 days to stabilize the diabetic condition.

### Foot ulceration

Induction of foot ulcer was based on an excisional model. After 14 days treatment of STZ injection, a glucometer was used to determine if each rat’s blood glucose level met the severe diabetes level of ≥ 300 mg/dL (hyperglycemia) by drawing blood from the tip of the tail. Rats with a blood glucose level greater than 300 mg/dL were used for foot ulceration. On the day of foot ulceration (Day 0), each rat was anesthetized by intraperitoneal injections of ketamine/xylazine cocktail. The skin surface of the right footpad was shaved and cleaned with 70% ethanol wipe. A 2 mm × 5 mm rectangular full thickness ulcer was created in the skin of the footpad on the right hind leg of each rat using a scalpel^72^.

### Treatment of DFU by nano-photodynamic therapy

The infected foot ulcers were irradiated daily in morning time for 1 week, starting on the 3rd day post infection induction. TBO–chit–Au–AgNPs was added in the middle and spread over the whole infected area. Thirty minutes after the addition, irradiation was carried out with 630 nm of laser light for 12 min and 50 s which corresponds to 100 J/cm^2^.

### Determination of bacterial load reduction (CFU/mL)

In order to confirm the absence of monomicrobial and polymicrobial *S. aureus* and *P. aeruginosa* colonization, bacterial load was measured for every sample by CFU counting on BHI agar plates and expressed as CFU per mL. Swab samples were collected from the infected foot surfaces of the untreated and treated rats starting on the 3rd, 6th and 9th day post treatment. Collected samples were serially diluted and plated onto BHI agar plates to determine the number of bacteria. The plates were incubated at 37 °C for 24 h before enumeration of colonies.

### Histopathological analysis

Fresh skin biopsies of control, untreated and treated rat groups were incised and fixed in 10% phosphate buffered formalin (pH 7.4). The tissues were then dehydrated in ascending grades of ethyl alcohol, cleared in xylene and mounted in molten paraplast at 58–62 °C. Thereafter, 5-μm histological sections were cut and stained with haematoxylin and eosin (H/E) to examine microorganism response^73^.

### Effects of nano-photodynamic therapy on growth factors and inflammatory cytokines involved in the pathogenesis of diabetic foot ulcers

To determine the production level of growth factors and inflammatory cytokines, blood samples were drawn under anesthesia and collected into sterile blood collection tubes from the eyes of controls rats, untreated rats, only TBO–chit–Au–AgNPs treated rats and TBO–chit–Au–AgNPs+ laser light treated rats. Thereafter, all rats were immediately sacrificed as per the guidelines. The samples were centrifuged at 5000 rpm for 5 min. Thereafter, the serum supernatant was aliquoted in microcentrifuge tubes and stored at − 80 °C for further analysis^74^.The commercially available enzyme-linked immunosorbent assay (ELISA) kits were used according to the manufacturers’ protocol: Rat TNF-α (Tumour Necrosis Factor-alpha) ELISA Kit (RAY BIOTECH, CAT: ELR-TNFΑ), Rat IL-6(Interleukin-6) ELISA Kit (RAY BIOTECH, CAT: ELR-IL6), Rat TGF-β1 (Transforming growth factor beta-1) ELISA Kit (ELABSCIENCE, CAT: E-EL-0162), Rat EGF (Epidermal growth factor) ELISA Kit (ELABSCIENCE, CAT: E-EL-R0369), Rat VEGF-A (Vascular endothelial growth factor-A) ELISA Kit (ELABSCIENCE, CAT: E-EL-R2603), Rat IGF-1 (Insulin-like growth factor-1) ELISA Kit (ELABSCIENCE, CAT: E-EL-R3001). The levels of TNF-α, IL-6, TGF-β1, EGF, VEGF-A and IGF-1 were measured by ELISA reader (BIORAD MICROPLATE READER, INDIA).

### Statistical analysis

All data were presented as averages of the values obtained ± S.D. of three independent experiments. Test groups were compared with the control group using the Student’s *t* test and one-way analysis of variance (ANOVA) was used for the comparison of multiple means^75^. For in vivo experiments, all the results were expressed as mean ± S.D. for four rats in each group. Data with *p* value of less than 0.05 (*p < 0.01, **p < 0.001, *** *p*< 0.0001) was considered statistically significant.

## Conclusion

We have developed a stable and biocompatible chitosan coated gold–silver core–shell nanoparticles conjugated with TBO. The synthesized TBO–chit–Au–AgNPs mediated photodynamic therapy was used for the first time to eliminate multi-drug resistant Gram-positive, and Gram-negative, monomicrobial as well as polymicrobial biofilms. Furthermore, this novel nano-phototheranostic complex proved as a nontoxic antibacterial agent to combat DFU caused by multi-drug resistant bacterial strains. Therefore, this approach holds a promising potential to be validated and implemented in clinical translation.

## Supplementary Information

Supplementary Information.
