# Supplementary material for: A nano phototheranostic approach of toluidine blue conjugated gold silver core shells mediated photodynamic therapy to treat diabetic foot ulcer
Source: Sci Rep. 2021 Dec 27;11:24464. doi: 10.1038/s41598-021-04008-x (PMC8712511; doi:10.1038/s41598-021-04008-x)
Supplement: Supplementary file 1 — Supplementary Information. [file 41598_2021_4008_MOESM1_ESM.docx]

**A nano phototheranostic approach of toluidine blue conjugated gold silver core shells mediated photodynamic therapy to treat diabetic foot ulcer**

Farheen Akhtar^1^, Asad U Khan^1^*, Bushra Qazi^1^, Senthilguru Kulanthaivel^2^, Prashant Mishra^2^, Kafil Akhtar^3^and Asif Ali^4^

^1^Medical Microbiology and Molecular Biology Lab., Interdisciplinary Biotechnology Unit,

Aligarh Muslim University, Aligarh, India.^2^Department of Biochemical Engineering and Biotechnology, Indian Institute of Technology, Delhi, India, ^3^ Department of Pathology, JNMC, A.M.U., Aligarh, ^4^ Department of Biochemistry, F/o Medicine, JNMC A.M.U., Aligarh.

**Running Title: Nanophotodynamic therapy against diabetic foot ulcer**

***Corresponding author:** Prof.Asad U. Khan,Medical Microbiology and Molecular Biology Lab., Interdisciplinary Biotechnology Unit, Aligarh Muslim University, Aligarh-202002, UP, India. Ph.: 0091-9837021912, Fax: 0091-571-2721776

Email id: asadukhan72@gmail.com

**SUPPLEMENTARY FILES**


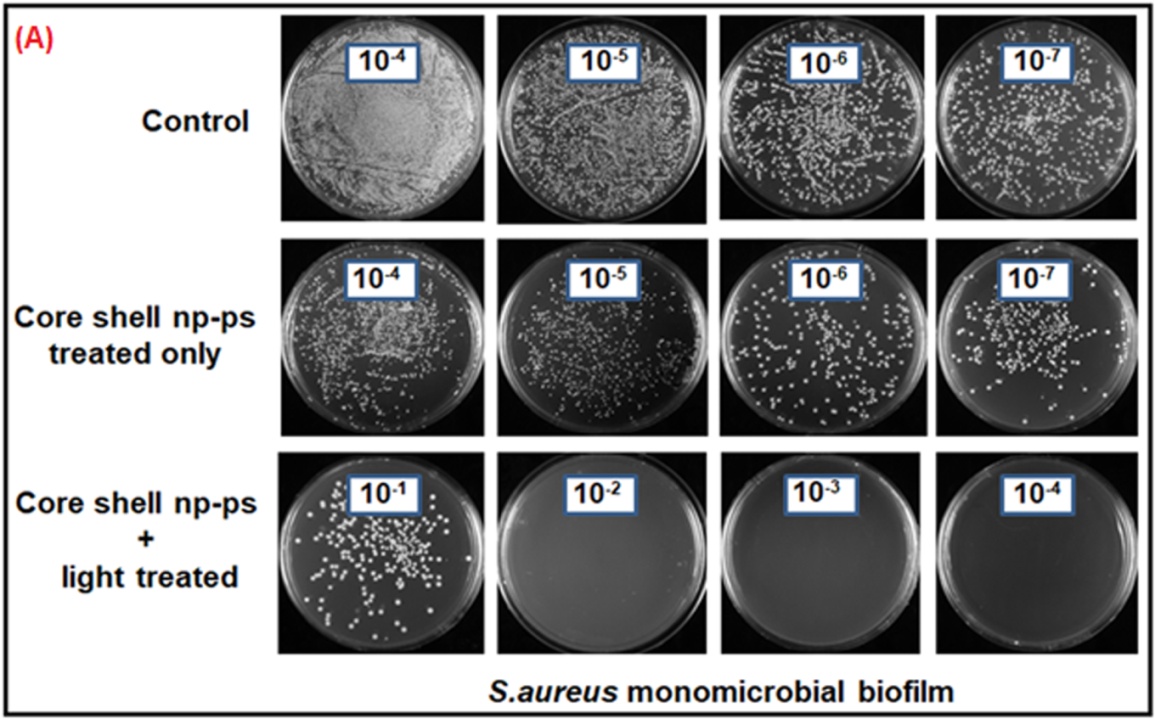


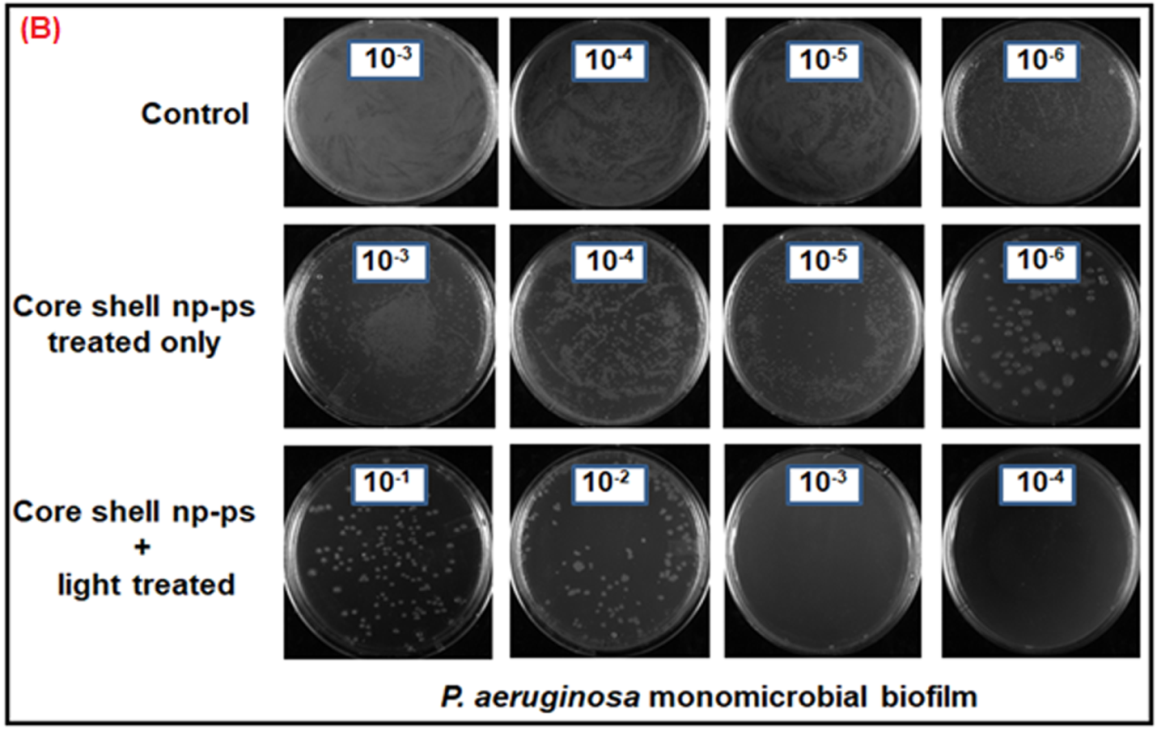


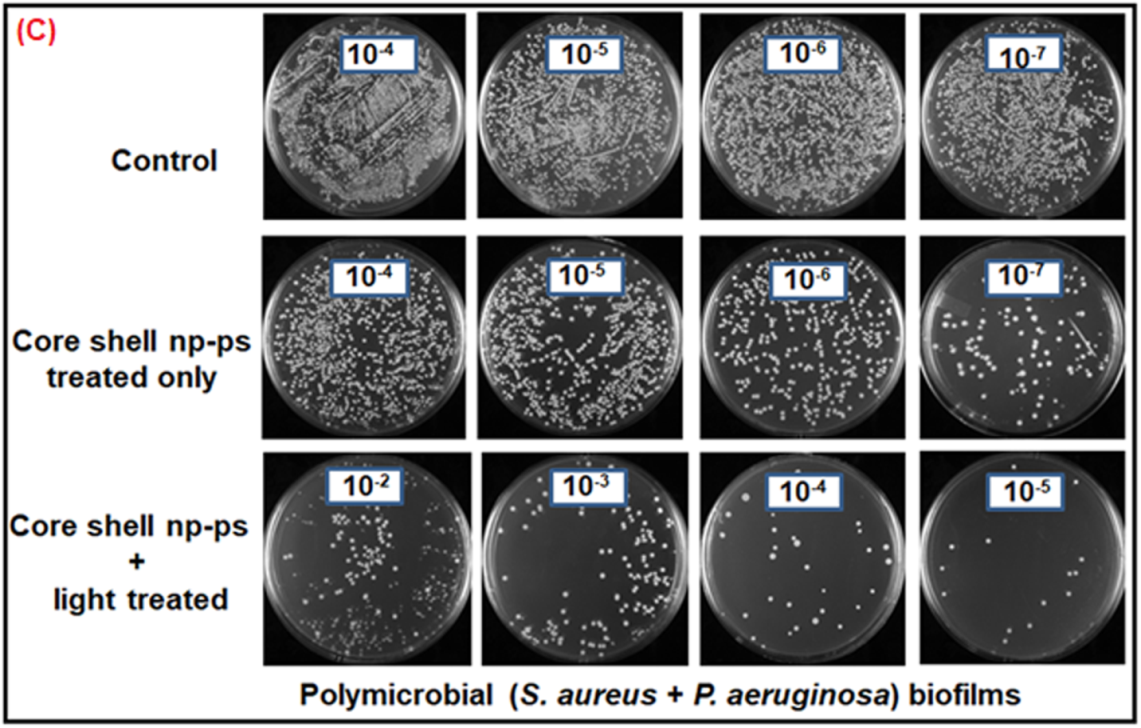


**Figure S1.In vitro colony formation:**Photographic image of (A) Monomicrobial*S.aureus*, (B) Monomicrobial*P.aeruginosa* and (C) Polymicrobial*S.aureus* + *P.aeruginosa*colonies after incubation with TBO-chit-Au-AgNPsor TBO-chit-Au-AgNPs followed by irradiation with 630 nm laser for 12 min and 50 seconds which corresponds to 100 Jcm^-2^  at 0.1300 W cm^-2^. Data are presented as mean ± SD (n = 3) and normalized to that of untreated control. Three replicates were performed for each experiment. Statistical significance was determined using one-way analysis of variance (p-value *p<0.01, **p<0.001, ***p<0.0001, ns=not significant).


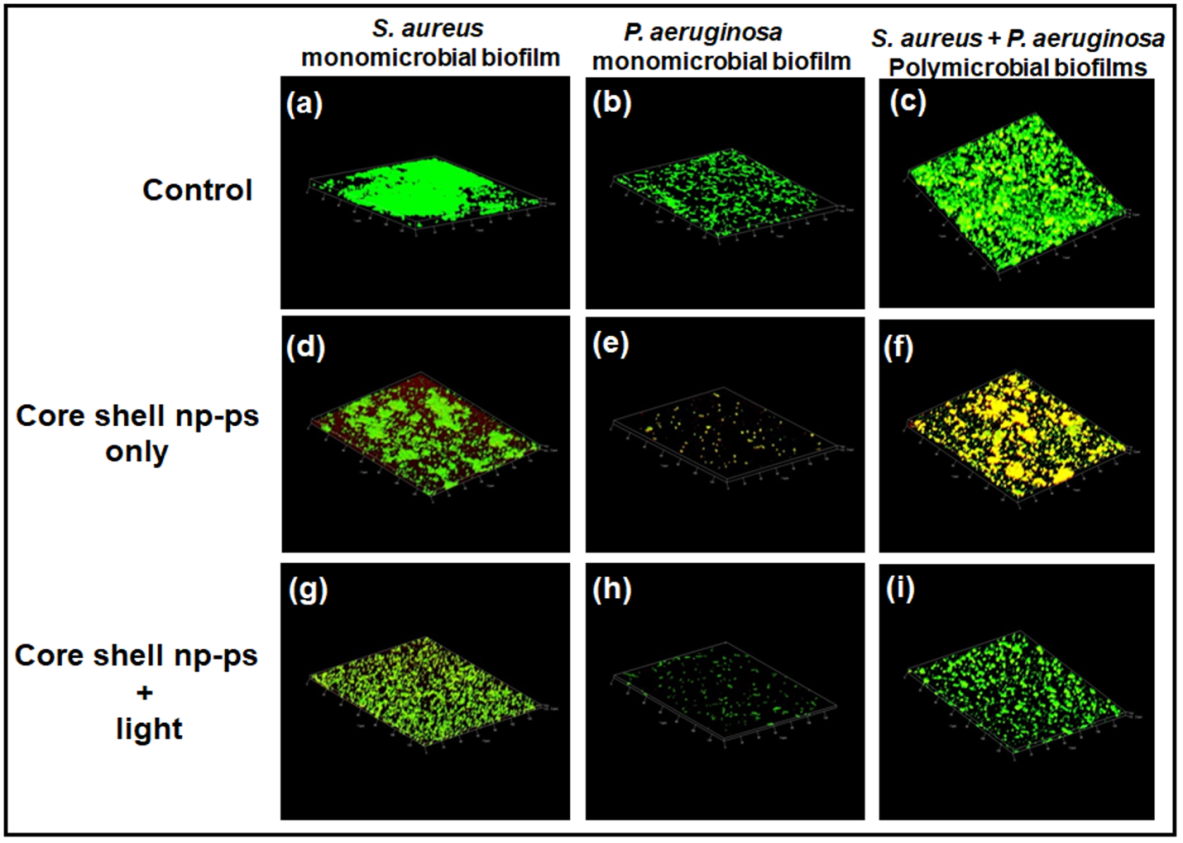


**Figure S2. Live/dead fluorescent staining images ofmonomicrobial*S.aureus*, monomicrobial*P.aeruginosa* and polymicrobial*S.aureus* + *P.aeruginosa*biofilms:** Representative fluorescence images of SYTO 9 (live, green) and PI (dead, red) stained bacteria in the groups of control (a)-(c), TBO-chit-Au-AgNPstreated (d)-(f) and TBO-chit-Au-AgNPs+laser treated (g)- (i) showing Z-depth of the corresponding samples.630 nm laser (100 Jcm^-2^, 12 min and 50 seconds) were used in the corresponding laser group. Scale bar = 10 μm.

**Table S1. The UV data of crystal violet assay**

**Table S2. The UV data of congo red assay**

**TableS3. Change in weight of the rats during the course of the experiment**

| S.No. | Groups | Weight of the rats (in grams) before the injection of Streptozotocin (STZ) | Weight of the rats (in grams) after the injection of Streptozotocin (STZ) | Weight of the rats  (in grams)  Day 3 | Weight of the rats  (in grams)  Day 6 | Weight of the rats  (in grams)  Day 9 |
| --- | --- | --- | --- | --- | --- | --- |
| 1. | Control  (normal rats) | 1. 280 2. 300 3. 270 4. 250 | 1. - 2. - 3. - 4. - | 1. - 2. - 3. - 4. - | 1. - 2. - 3. - 4. - | 1. - 2. - 3. - 4. - |
| 2. | Diabetic without ulcer  (untreated rats) | 1. 275 2. 290 3. 270 4. 250 | 1. 274 2. 288 3. 268 4. 248 | 1. 273.5 2. 286 3. 266.5 4. 247.5 | 1. 273 2. 285.5 3. 265 4. 246.2 | 1. 272.8 2. 285 3. 264.9 4. 246 |
| 3. | *Pseudomonas aeruginosa*  Only ulcer  (untreated rats) | 1. 256 2. 280 3. 268 4. 276 | 1. - 2. - 3. - 4. - | 1. - 2. - 3. - 4. - | 1. - 2. - 3. - 4. - | 1. - 2. - 3. - 4. - |
| 4. | *Pseudomonas aeruginosa*  Diabetic with ulcer  (untreated rats) | 1. 285 2. 260 3. 295 4. 298 | 1. 283 2. 258.5 3. 293.6 4. 297.5 | 1. 282.5 2. 257.9 3. 292 4. 296.7 | 1. 282 2. 257.8 3. 291.5 4. 296 | 1. 282 2. 256.9 3. 291 4. 296 |
| 5. | *Pseudomonas aeruginosa* Diabetic with ulcer (TBO-chit-Au-AgNPs treated rats) | 1. 250 2. 280 3. 252 4. 274 | 1. 248 2. 278.5 3. 251.1 4. 273.5 | 1. 247.2 2. 278 3. 250.9 4. 272.4 | 1. 246 2. 277.2 3. 249.8 4. 271.5 | 1. 245.2 2. 276 3. 249.2 4. 270.6 |
| 6. | *Pseudomonas aeruginosa*  Diabetic with ulcer (TBO-chit-Au-AgNPs+light treated rats) | 1. 250 2. 292 3. 274 4. 281 | 1. 248.7 2. 291.2 3. 272.1 4. 280.6 | 1. 247.4 2. 290 3. 271.3 4. 278.6 | 1. 246.9 2. 289.1 3. 270.2 4. 277.5 | 1. 246 2. 288.9 3. 269.8 4. 276.4 |
| 7. | *Staphylococcus aureus*  Only ulcer  (untreated rats) | 1. 287 2. 275 3. 256 4. 290 | 1. - 2. - 3. - 4. - | 1. - 2. - 3. - 4. - | 1. - 2. - 3. - 4. - | 1. - 2. - 3. - 4. - |
| 8. | *Staphylococcus aureus*  Diabetic with ulcer  (untreated rats) | 1. 294 2. 285 3. 276 4. 259 | 1. 292.8 2. 283.5 3. 274.9 4. 257.4 | 1. 291.5 2. 281.9 3. 272.5 4. 257 | 1. 290.4 2. 280.5 3. 271.1 4. 255.9 | 1. 289.9 2. 280 3. 270.8 4. 254.7 |
| 9. | *Staphylococcus aureus*  Diabetic with ulcer  (TBO-chit-Au-AgNPs treated rats) | 1. 265 2. 290 3. 286 4. 274 | 1. 262.9 2. 287.5 3. 285 4. 272.4 | 1. 261.5 2. 286.9 3. 283.4 4. 271 | 1. 260.6 2. 286 3. 282.7 4. 269.9 | 1. 260.2 2. 285.3 3. 282 4. 269 |
| 10. | *Staphylococcus aureus*  Diabetic with ulcer (TBO-chit-Au-AgNPs+light treated rats) | 1. 290 2. 265 3. 300 4. 298 | 1. 287.9 2. 264.1 3. 298.2 4. 297.5 | 1. 287 2. 263.5 3. 297 4. 297 | 1. 286.2 2. 263 3. 296.4 4. 295.9 | 1. 286 2. 262.5 3. 295.8 4. 294.7 |
| 11. | Polymicrobial (*Staphylococcus aureus* + *Pseudomonas aeruginosa*)  Only ulcer  (untreated rats) | 1. 300 2. 298 3. 269 4. 292 | 1. - 2. - 3. - 4. - | 1. - 2. - 3. - 4. - | 1. - 2. - 3. - 4. - | 1. - 2. - 3. - 4. - |
| 12. | Polymicrobial (*Staphylococcus aureus* + *Pseudomonas aeruginosa*)  Diabetic with ulcer (untreated rats) | 1. 270 2. 265 3. 282 4. 290 | 1. 268.2 2. 263.9 3. 281.1 4. 289 | 1. 268 2. 261.7 3. 280 4. 288.3 | 1. 267.4 2. 261 3. 278.7 4. 287.9 | 1. 266.5 2. 259.4 3. 277.9 4. 286.5 |
| 13. | Polymicrobial (*Staphylococcus aureus* + *Pseudomonas aeruginosa*)  Diabetic with ulcer (TBO-chit-Au-AgNPs treated rats) | 1. 285 2. 298 3. 259 4. 278 | 1. 283.1 2. 296.7 3. 257.9 4. 277.4 | 1. 282.8 2. 296 3. 256.2 4. 276.9 | 1. 281.7 2. 295.8 3. 256 4. 276.2 | 1. 281 2. 294.7 3. 255.4 4. 275.9 |
| 14. | Polymicrobial (*Staphylococcus aureus* + *Pseudomonas aeruginosa*)  Diabetic with ulcer (TBO-chit-Au-AgNPs+light treated rats) | 1. 290 2. 275 3. 270 4. 268 | 1. 288.9 2. 274.4 3. 268.3 4. 266.7 | 1. 288.4 2. 273.2 3. 267 4. 265.2 | 1. 288 2. 272.9 3. 265.8 4. 264.7 | 1. 287.6 2. 272 3. 265.4 4. 264.1 |

**Supplementary Table S4. *In-vivo* bacterial load reduction (CFU/ml)**

1. **CFU/ml at day 3 post ulceration**

|  | **Ulcer (Untreated)** | **Diabetic+Ulcer (Untreated)** | **Core shell np-ps treated** | **Core shell np-ps + light treated** |
| --- | --- | --- | --- | --- |
| ***S. aureus*** | 4.27×10^9^ | 5.04×10^9^ | 3.32×10^9^ | 1.42×10^9^ |
| ***P. aeruginosa*** | 3.51×10^9^ | 4.41×10^9^ | 2.53×10^9^ | 3.7×10^8^ |
| ***Polymicrobial (S. aureus+P.aeruginosa)*** | 6.38×10^9^ | 5.59×10^9^ | 4.28×10^9^ | 3.99×10^9^ |

1. **CFU/ml at day 6 post ulceration**

|  | **Ulcer (Untreated)** | **Diabetic+Ulcer (Untreated)** | **Core shell np-ps treated** | **Core shell np-ps + light treated** |
| --- | --- | --- | --- | --- |
| ***S.aureus*** | 3.97×10^8^ | 4.52×10^8^ | 2.58×10^8^ | 5×10^7^ |
| ***P.aeruginosa*** | 7.6×10^7^ | 3.4×10^8^ | 4.1×10^7^ | 1.3×10^7^ |
| ***Polymicrobial (S.aureus+P.aeruginosa)*** | 3.6×10^8^ | 4.32×10^8^ | 2.25×10^8^ | 1.16×10^8^ |

1. **CFU/ml at day 9 post ulceration**

|  | **Ulcer (Untreated)** | **Diabetic+Ulcer (Untreated)** | **Core shell np-ps treated** | **Core shell np-ps + light treated** |
| --- | --- | --- | --- | --- |
| ***S.aureus*** | 4.71×10^7^ | 2.93×10^7^ | 9.3×10^6^ | 7.5×10^6^ |
| ***P.aeruginosa*** | 1.24×10^7^ | 2.98×10^7^ | 5.8×10^6^ | 0 |
| ***Polymicrobial (S.aureus+P.aeruginosa)*** | 1.08×10^7^ | 4.76×10^7^ | 1.6×10^7^ | 5.4×10^6^ |
